# Structural basis of trehalose recognition by the mycobacterial LpqY-SugABC transporter

**DOI:** 10.1016/j.jbc.2021.100307

**Published:** 2021-01-19

**Authors:** Christopher M. Furze, Ignacio Delso, Enriqueta Casal, Collette S. Guy, Chloe Seddon, Chelsea M. Brown, Hadyn L. Parker, Anjana Radhakrishnan, Raul Pacheco-Gomez, Phillip J. Stansfeld, Jesus Angulo, Alexander D. Cameron, Elizabeth Fullam

**Affiliations:** 1 School of Life Sciences, University of Warwick, Coventry, UK; 2 Instituto de Síntesis Química y Catálisis Homogénea (ISQCH), Universidad de Zaragoza, CSIC, Zaragoza, Spain; 3 School of Pharmacy, University of East Anglia, Norwich, Norfolk, UK; 4 Malvern Panalytical Ltd, Malvern, UK; 5 Department of Chemistry, University of Warwick, Coventry, UK; 6 Departamento de Química Orgánica, Universidad de Sevilla, Sevilla, Spain; 7 Instituto de Investigaciones Químicas (CSIC-US), Sevilla, Spain

**Keywords:** *Mycobacterium tuberculosis*, trehalose, carbohydrate, ABC transporter, LpqY-SugABC transporter, structural biology, structure-function, ABC, ATP-binding cassette, DEEP-STD NMR, differential epitope mapping STD NMR, ITC, isothermal titration calorimetry, MD, molecular dynamic, MST, microscale thermophoresis, *Mtb*, *Mycobacterium tuberculosis*, *Mtr*, *Mycobacterium thermoresistible*, STD NMR, saturation transfer difference NMR, TB, tuberculosis, *T_m_*, melting temperature

## Abstract

The *Mycobacterium tuberculosis* (*Mtb*) LpqY-SugABC ATP-binding cassette transporter is a recycling system that imports trehalose released during remodeling of the *Mtb* cell-envelope. As this process is essential for the virulence of the *Mtb* pathogen, it may represent an important target for tuberculosis drug and diagnostic development, but the transporter specificity and molecular determinants of substrate recognition are unknown. To address this, we have determined the structural and biochemical basis of how mycobacteria transport trehalose using a combination of crystallography, saturation transfer difference NMR, molecular dynamics, site-directed mutagenesis, biochemical/biophysical assays, and the synthesis of trehalose analogs. This analysis pinpoints key residues of the LpqY substrate binding lipoprotein that dictate substrate-specific recognition and has revealed which disaccharide modifications are tolerated. These findings provide critical insights into how the essential *Mtb* LpqY-SugABC transporter reuses trehalose and modified analogs and specifies a framework that can be exploited for the design of new antitubercular agents and/or diagnostic tools.

Tuberculosis (TB), caused by the bacterial pathogen *Mycobacterium tuberculosis* (*Mtb*), is now the leading cause of death from a single infectious agent world-wide claiming over 1.5 million lives each year (https://www.who.int/teams/global-tuberculosis-programme/tb-reports). *Mtb* is a highly successful intracellular pathogen, which has co-evolved over thousands of years to enable it to adapt within the human host and develop highly effective strategies to persist and survive (1). To thrive within this nutrient-restricted host environment, *Mtb* must access scarce energy sources; however, the precise nutritional requirements of *Mtb* and the mechanisms of assimilation are poorly understood (2, 3). Unraveling the processes and transporters in *Mtb* involved in nutrient scavenging and the import of these critical energy sources should lead to new intervention strategies to combat this major global pathogen.

For many pathogens, carbohydrates are critical carbon sources for the production of energy and essential biomolecules, which are required for a wide range of cellular processes. However, the diversity and availability of sugars to *Mtb* during infection remain largely unclear (2, 3). Trehalose (α-D-glucopyranosyl-α-D-glucopyranoside, α,α-trehalose) is an unusual nonmammalian disaccharide that is highly abundant in mycobacteria (4). Trehalose-containing glycolipids are major components of the mycobacterial cell envelope that contribute to the virulence of the *Mtb* pathogen and provide an extracellular source of “free” trehalose which can be used as a carbon and energy source (5, 6, 7). Trehalose is released either through the hydrolysis of trehalose-containing glycolipids by serine esterases or during the assembly of the mycobacterial cell envelope mediated by the antigen 85 complex (8, 9, 10). Recent studies in mutant strains of *Mtb* have demonstrated that the LpqY-SugABC (Rv1235-Rv1238) ATP-binding cassette (ABC) transporter recognizes trehalose and enables the recovery and recycling of this liberated cell wall disaccharide that would otherwise be lost (6). Mutants of *Mtb* that lack functional components of the LpqY-SugABC importer are attenuated in mice infection models demonstrating the critical importance of trehalose uptake for *Mtb* virulence (6). Given that trehalose import is fundamental for virulence and essential for *Mtb* to survive, the *Mtb* trehalose transporter is an attractive target for inhibitor design. Despite the importance of trehalose uptake in mycobacteria, the molecular details that govern how this disaccharide are recognized and whether alternative sugars are substrates for this recycling system remain unresolved. Some understanding into the substrate preference of this mycobacterial ABC-transporter can be obtained from studies which found that modified trehalose analogs retaining the α1-1-glycosidic linkage are actively imported by the LpqY-SugABC recycling system and metabolically incorporated into the trehalose-mycolates located within the cell envelope (11, 12, 13). Whether the mycobacterial LpqY-SugABC transporter is able to facilitate the import of alternative, more diverse, sugars is not yet known.

Here, we have used a combination of chemical, biochemical, and biophysical approaches to describe the functional and structural characterization of the mycobacterial LpqY substrate binding domain of the LpqY-SugABC ABC transporter and reveal its substrate specificity and the molecular framework that underpins the recognition of trehalose and related substrates. These findings offer fundamental insights into how mycobacteria recognize and import trehalose, a critical process in virulence and survival of the *Mtb* pathogen.

## Results

### Production of Mtr LpqY

The optimal conditions for the production of *Mtb* LpqY and mycobacterial LpqY homologs were explored extensively in *Escherichia coli*. This yielded LpqY from *Mycobacterium thermoresistible* (*Mtr*), which has high sequence identity (72%) to *Mtb* LpqY at the amino acid level (Fig. S1). Soluble *Mtr* LpqY protein was readily obtained and purified using Ni^2+^-affinity and size-exclusion chromatography (Fig. S2), and the identity of the *Mtr* LpqY protein was confirmed by mass spectrometry.

### Substrate specificity of Mtr LpqY

**Figure 1:**
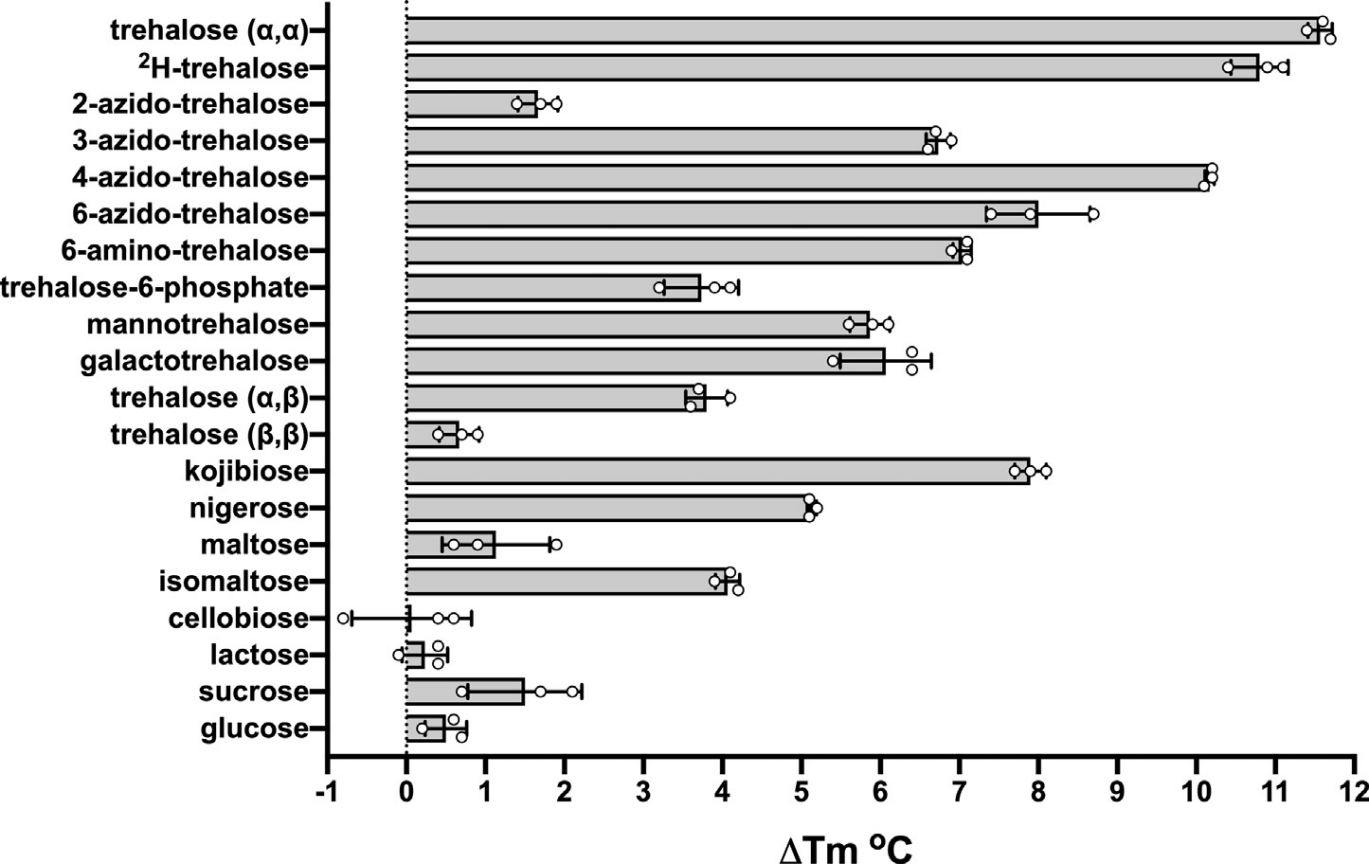
**Thermal shift assay probing a panel of potential *Mtr* LpqY ligands**. Bar graph illustrating the Δ*T*_m_ shift of *Mtr* LpqY for a series of carbohydrates (10 mM). Data shown are from three independent repeats; error bars represent ± standard deviation. The substrate structures are shown in Fig. S4. The *T*_*m*_ of *Mtr* LpqY is 66.0 °C ± 0.2 deg. C. *Mtr*, *Mycobacterium thermoresistible*.

To establish whether the LpqY-SugABC transporter is specific for trehalose or is instead promiscuous for other carbohydrates, a panel of monosaccharides and disaccharides (10 mM) were screened for their ability to stabilize the melting temperature (*T*_*m*_) of the *Mtr* LpqY substrate binding domain. In total, 62 potential substrates were probed, and the observed change in the melting temperature (*ΔT*_*m*_) of *Mtr* LpqY was assessed, which can be indicative of binding (Fig. 1, Figs. S3 and S4). Notably, trehalose resulted in the highest thermal shift (*ΔT*_*m*_ 11.5 °C) of *Mtr* LpqY relative to the protein alone and compared with all substrates tested, indicating that *Mtr* LpqY has a clear preference for this physiologically relevant sugar (Fig. S3).

Next, we probed whether modified trehalose analogs known to be imported by mycobacteria, including ^2^H-trehalose, 2-azido-2-deoxy-α,α′-trehalose (2-azido-trehalose), 4-azido-4-deoxy-α,α′-trehalose (4-azido-trehalose), 6-azido-6-deoxy-α,α′-trehalose (6-azido-trehalose), α-D-mannopyranosyl-(1→1)-α-D-glucopyranoside (mannotrehalose), and α-D-galactopyranosyl-(1→1)-α-D-glucopyranoside (galactotrehalose) (see SI methods for synthetic details), are substrates of *Mtr* LpqY (11, 12, 13, 14). All of these disaccharides influenced the melting temperature of *Mtr* LpqY but to a lesser extent (Fig. 1). In addition to 6-azido-trehalose, we tested 6-amino-6-deoxy-α,α′-trehalose (6-amino-trehalose) and trehalose-6-phosphate to probe whether alternative moieties at this position are tolerated. The amino-group has a similar *ΔT*_*m*_ shift to 6-azido-trehalose, whereas the larger phosphate group had less impact. To determine the importance of the azide-group position, we tested 2-, 3-, 4- and 6-azido trehalose analogs. 4-azido-trehalose had the highest *ΔT*_*m*_ shift, which is comparable to trehalose. 6-azido-trehalose and 3-azido-trehalose had *ΔT*_*m*_ shifts in a similar range, whereas 2-azido-trehalose showed only a minor shift. Thus, azide-trehalose analogs are recognized but to different extents. We then asked if *Mtr* LpqY recognizes other sugars. This analysis highlighted the importance of the stereochemistry of the α1,1-glycosidic bond in substrate recognition as there was a reduction in recognition of α,β-trehalose, with a αβ1-1 linkage, and almost complete loss of recognition of β,β-trehalose where the two glucose units are orientated through a β1-1 glycosidic bond. Furthermore, the regiochemistry of the glycosidic bond is crucial for substrate recognition and replacement of the preferred α1,1 linkage with either an α1-2 (kojibiose), α1-3 (nigerose), α1-4 (maltose), or α1-6 (isomaltose) glycosidic bond resulted in reduced *ΔT*_*m*_ shifts (Fig. 1). In marked contrast, all of the other disaccharides and monosaccharides evaluated, including the glucose monosaccharide subunit of trehalose, had no influence on the *ΔT*_*m*_ indicating that these sugars are unlikely to be substrates (Fig. S3). It is particularly noteworthy that the single glucose unit is not recognized in these assay conditions, suggesting that a disaccharide is required for substrate binding and recognition to occur.

To validate our findings from the thermal shift screening the binding interactions of *Mtr* LpqY were assessed with the disaccharide substrates that resulted in the largest thermal shift. Isothermal titration calorimetry (ITC) experiments were performed with the preferred trehalose substrate and also with galactotrehalose, which was found to result in a ∼2-fold reduction in the *ΔT*_*m*_ of *Mtr* LpqY compared with trehalose. ITC analysis revealed a 1:1 binding stoichiometry for both sugars:*Mtr* LpqY and equilibrium dissociation constants (*K*_*d*_) of 1.1 ± 0.04 μM and 2.1 ± 0.2 μΜ respectively (Fig. S5). This is in agreement with the range of reported *K*_*d*_ values determined by ITC for substrate binding domains of other ABC transporters (15, 16), with a *K*_*d*_ value of 13 μM reported for an α-glycoside ABC transporter from *Thermus thermophilus* (17). This provides direct evidence that this mycobacterial importer is a high-affinity trehalose transporter. We also tested the binding affinity of trehalose by microscale thermophoresis (MST) and confirmed that binding is also in the micromolar range, with an observed *K*_*d*_ value of 72 μM (Table 1, Fig. S6). Given that MST consumes significantly less protein than ITC, we therefore used the MST assay to evaluate the binding affinities of the other sugar substrates. The *K*_*d*_ values obtained are reported in Table 1. Among all of the substrates tested, we were able to determine binding affinities for ^2^H-trehalose, 2-, 3-, 4- and 6-azido-trehalose, galactotrehalose, mannotrehalose, and kojibiose. As expected, the *K*_*d*_ value for the deuterated ^2^H-trehalose analog was comparable to trehalose, whereas the modified trehalose derivatives displayed slightly weaker binding affinities. This is consistent with the use of azido-modified trehalose tools developed to evaluate trehalose metabolism in mycobacteria (13).

**Table 1:** Binding data for *Mtr* LpqY

Substrate	*K*_*d*_ (μM)	*ΔT*_*m*_
Trehalose	72.1 ± 3.1	11.6 ± 0.2
^2^H-trehalose	120.2 ± 8.2	10.8 ± 0.4
2-azido-2-deoxy-trehalose	1915.6 ± 7.7	1.7 ± 0.1
3-azido-3-deoxy-trehalose	474.9 ± 17.2	6.7 ± 0.2
4-azido-4-deoxy-trehalose	246.6 ± 7.1	10.2 ± 0.1
6-azido-6-deoxy-trehalose	442.7 ± 5.3	8.0 ± 0.7
6-amino-6-deoxy-trehalose	---	7.0 ± 0.1
Trehalose-6-phosphate	---	3.7 ± 0.2
Galactotrehalose	236.9 ± 3.3	6.1 ± 0.6
Mannotrehalose	798.9 ± 48.8	5.9 ± 0.3
Kojibiose	2353.4 ± 122.7	7.9 ± 0.2
Nigerose	---	5.1 ± 0.1
Isomaltose	---	4.1 ± 0.2
α,β-trehalose	---	3.7 ± 0.1

*Mtr*, *Mycobacterium thermoresistible*.

“---” result was ambiguous because of low signal to noise ratios, and reliable *K*_*d*_ values were unable to be determined at concentrations >10 mM. Mean ± SEM are from at least three independent experiments.

In these studies, we observed that the asymmetric epimeric analogs, galactotrehalose and mannotrehalose, showed an ∼3 and ∼12-fold reduction in binding affinity respectively. These findings are compatible with our recent studies in *Mycobacterium smegmatis* which, unexpectedly, showed that 6-azido-galactotrehalose is incorporated into the mycobacterial cell envelope with a similar efficiency as 6-azido-trehalose *via* the *M. smegmatis* LpqY-SugABC transporter (12, 13). This result indicates that *Mtr* LpqY is able to tolerate epimerization of the hydroxy group at the 4-position, whereas epimerization at the 2-position is less favored. The preference for the α1-1 glycosidic bond was further confirmed through evaluation of alternative α-glycoside disaccharides. The binding affinity for these analogs could only be determined for kojibiose (α1-2) under these assay conditions with a ∼30-fold increase in the *K*_*d*._ value observed. We were unable to obtain reliable *K*_*d*_ values for nigerose and isomaltose because of low signal to noise ratios, suggesting that sugars with α1-3 and α1-6 glycosidic bonds have reduced binding affinities and are not recognized. Finally, we did not observe binding to glycerophosphocholine which is the substrate of the *Mtb* UgpABCE ABC transporter (Fig. S3) (18), indicating that each *Mtb* carbohydrate importer has a distinct substrate preference and are only able to accept minor structural modifications (18, 19). Taken together, these data establish that the *Mtr* LpqY substrate binding protein is highly specific for trehalose.

### Co-crystal structure of Mtr LpqY with trehalose

**Figure 2:**
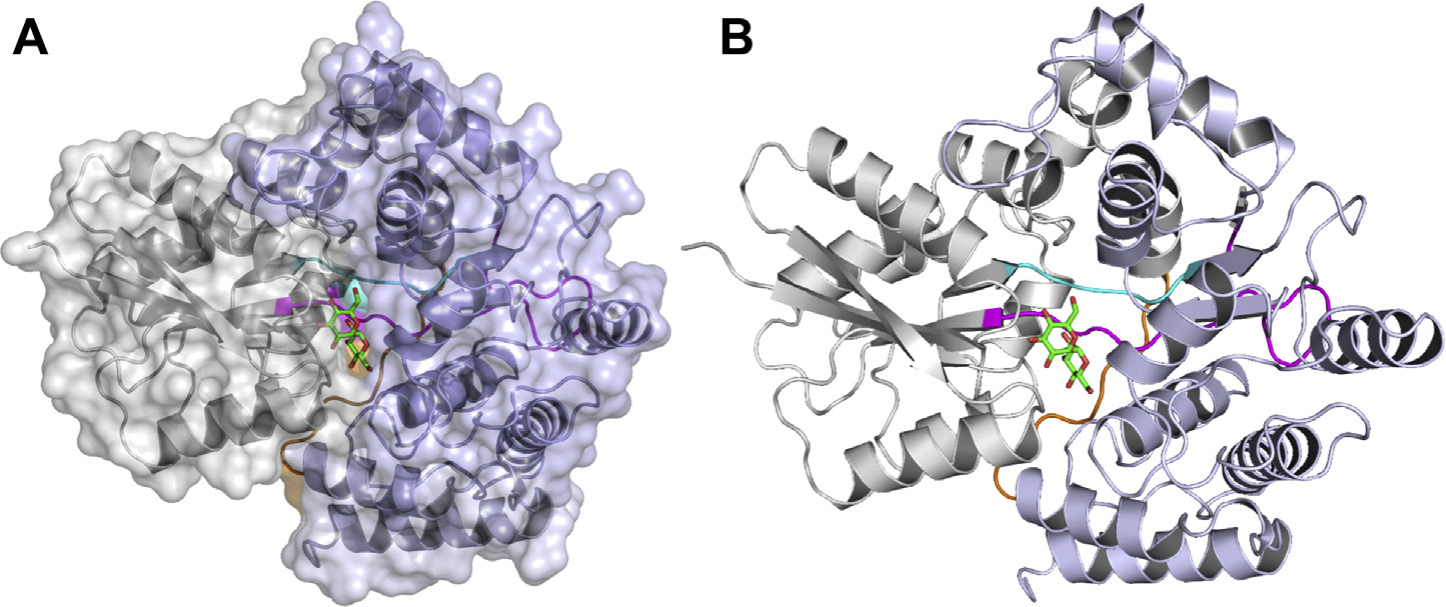
**Crystal structure of *Mtr* LpqY in complex with trehalose.***A*, surface representation of *Mtr* LpqY in complex with trehalose. *B*, cartoon representation of *Mtr* LpqY in complex with trehalose. The two domains and the hinge regions are highlighted: domain I (*gray*) and domain II (*blue*); loop 1 (*cyan*), loop 2 (*magenta*), loop 3 (*orange*). The trehalose ligand is represented as sticks with *green carbon atoms*. *Mtr*, *Mycobacterium thermoresistible*.

To determine the molecular and structural basis of trehalose recognition, we solved the crystal structure of *Mtr* LpqY with the trehalose substrate present (Fig. 2). The *Mtr* LpqY-trehalose complex crystallized in space group P4_1_2_1_2_1_, and the structure was determined by exploiting the anomalous signal from iodide-soaked crystals. The model containing bound iodine ions was then used as a search model to solve the structure of a native, higher-resolution, data set by molecular replacement, and the final *Mtr* LpqY-trehalose complex structure was refined at a resolution of 1.7 Å to an *R*_work_ of 16.9% and *R*_free_ of 19.4% (see Table S1 for the data collection and refinement statistics). Two *Mtr* LpqY protein molecules are present within the asymmetric unit. Structural superposition indicates that each subunit is equivalent, aligning with a r.m.s.d. of 0.45 Å over all residues, whereas crystal packing and analysis of the crystal packing interfaces indicates that *Mtr* LpqY does not form dimers or higher oligomers (20, 21). This is consistent with our solution size-exclusion studies where *Mtr* LpqY is found as a monomer (Fig. S2*D*). Therefore, it is likely that the monomer is the biologically relevant unit, which is consistent with the known oligomeric state of substrate binding domains from other ABC transporters (22, 23).

### Overall structure of the Mtr LpqY-trehalose complex

The overall architecture of *Mtr* LpqY is typical of substrate-binding domains of ABC transporters, consisting of two globular α/β domains joined by a hinge region (23), which in this instance is formed from three flexible loops. Domain I (residues 14–132 and 335–400) and domain II (residues 137–317 and 409–448) both comprise of a central β-sheet that is flanked on both sides by α-helices. The two lobes are connected *via* three flexible loops: Thr132-Leu137 (loop 1), Ala317-Leu335 (loop 2), and Asn400-Val409 (loop 3). The *Mtr* LpqY-trehalose complex adopts a closed conformation, which is further stabilized through a central arginine residue within the hinge region (Arg404, loop 3) that forms interdomain hydrogen bonds with the carbonyl oxygen of Leu332 located on loop 2 and the carboxylate of Glu179 from domain 2 as well as being directly involved with substrate binding.

### Ligand binding site of Mtr LpqY

**Figure 3:**
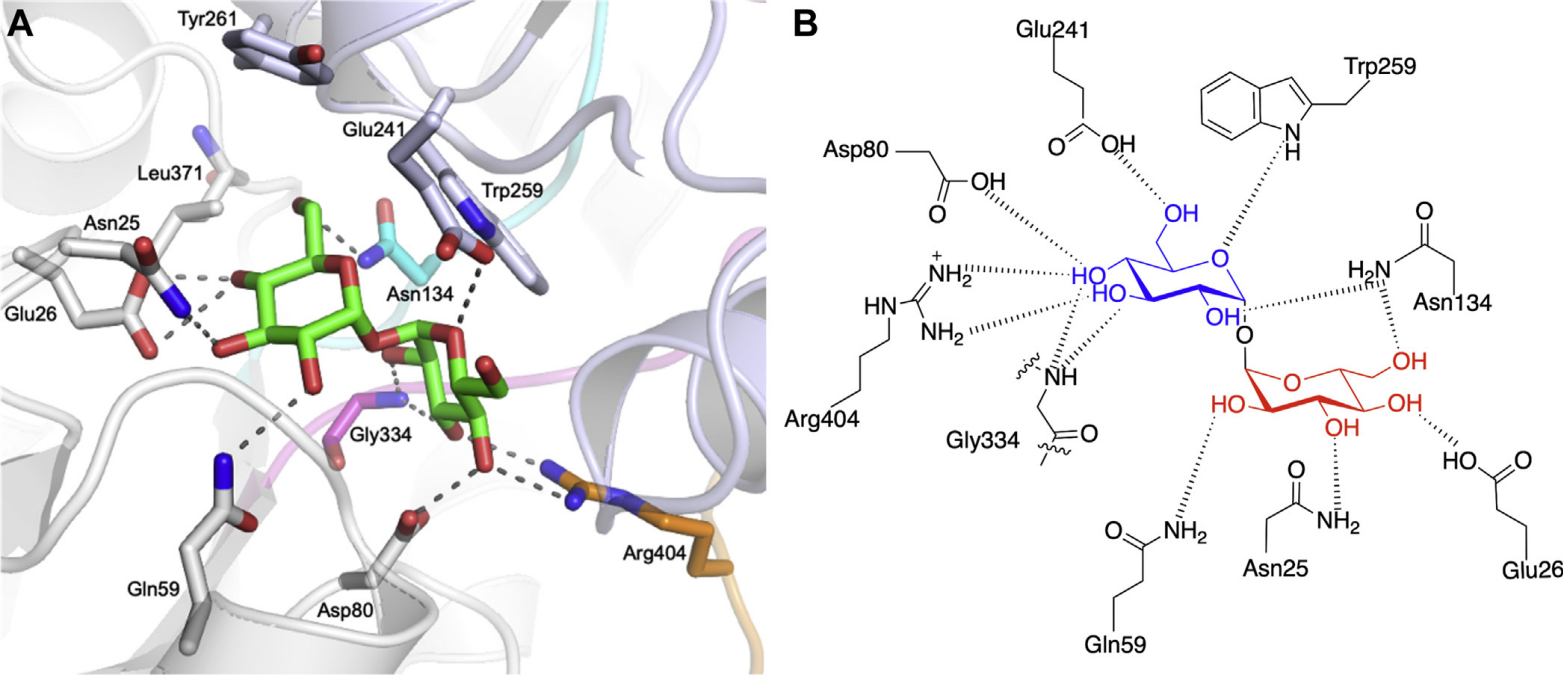
**Trehalose binding site in *Mtr* LpqY.***A*, illustration of the *Mtr* LpqY trehalose binding site showing trehalose and the interacting residues in stick representation. Domain I (*gray*) and domain II (*blue*); loop 1 (*cyan*), loop 2 (*magenta*), loop 3 (*orange*). The trehalose ligand is represented as sticks with *green carbon atoms*. *B*, schematic of trehalose interactions with *Mtr* LpqY. *Dashed lines* represent hydrogen bonding. Glc-1 (*blue*), Glc-2 (*red*). *Mtr*, *Mycobacterium thermoresistible*.

The trehalose molecule was clearly defined in the electron density and resides within the acidic binding cleft formed between domains I and II and interacts with residues from both domains (Fig. 2, Fig. S7). Trehalose comprises two glucopyranosyl units connected by a α1,1-glycosidic bond. In the *Mtr* LpqY structure, both of the glucose rings adopt a classical ^4^C_1_ chair conformation with almost equivalent dihedral angles across the glycosidic bonds (*φ*_H_: 63.2°, *ψ*_H_ 65.4°), thus having rotational symmetry about the central glycosidic oxygen atom, mimicking the conformation of anhydrous trehalose crystallized in the absence of protein (24). In *Mtr* LpqY, trehalose is orientated such that one glucose molecule (Glc-1) is buried at the base of the binding cleft in close proximity to the hinge-region containing Arg404, whereas the second glucose molecule (Glc-2) extends outwards toward the entrance of the binding channel (Fig. 3).

The disaccharide is anchored into place through a significant network of hydrogen bonds in which all sugar hydroxyl groups participate, with additional hydrogen bond interactions formed through the ring oxygen of Glc-1 as well as the glycosidic oxygen atom. The buried Glc-1 molecule is orientated to interact with the side chains of Asp80, Asn134, Glu241, Trp259, Arg404, and with the backbone amide of Gly334. The second glucose molecule (Glc-2) is stabilized through direct hydrogen bond interactions with Asn25, Glu26, Gln59, and Asn134. Water interactions are also observed with both C6-hydroxyl groups, the glycosidic oxygen atom as well as an intraglucose bridging water between the C6-hydroxyl group of Glc-1 and the C2-hydroxyl group of Glc-2. Hydrophobic interactions provide additional stabilization between the indole side chain of Trp259 and Glc-1, with potential van der Waals interactions between the side chains of Trp261 and Leu371 with Glc-2. Recognition of trehalose appears to be largely driven through accommodation of this ligand within a binding pocket where substrate selectivity is underpinned through an extensive hydrogen bonding network. These interactions have a pronounced effect on substrate recognition and dictate the stringent stereoselective requirement for an α1,1-linked disaccharide. All of the residues that interact with trehalose are conserved in *Mtb* LpqY with the exception of residues Asn25 and Glu26 that are located on a short-loop region comprising four residues formed between β1 and α1. In *Mtb*, Asn25 and Glu26 are instead replaced with a threonine and an aspartic acid residue, respectively, so while the residues are slightly smaller, the properties of the side chains are maintained. Evaluating the sequence alignment of mycobacterial LpqY homologs reveals a much greater sequence divergence among these nonconserved loop residues, suggesting a degree of flexibility of substrate recognition in this region, though an acidic residue is always found at position 26 (Fig. S8).

### Site-directed mutagenesis of Mtr LpqY

To complement our structural studies and understand the functional importance of residues coordinating trehalose within the *Mtr* LpqY binding pocket point mutations were introduced to substitute nine individual residues to an alanine (Table 2). Two further residues, Asp25 and Glu26, in the short loop region that interacts with Glc-2, were mutated to threonine and aspartic acid, respectively, to replicate the *Mtb* LpqY binding-site. Proper folding was assessed by circular dichroism, and we determined these mutations were not detrimental to correct folding except for the Asp80Ala mutant, which was inherently less stable, and had a distinctive circular dichroism profile (Fig. S9). The corresponding aspartic acid residue (Asp70) in *T. thermophilus* has been implicated in enabling the closure of domains I and II upon substrate binding, which may explain the instability of this particular *Mtr* LpqY mutant (17). The MST was used to determine the binding affinities of each site-directed *Mtr* LpqY mutant protein with trehalose. Complete abrogation of binding was observed when Glu241, Trp259, and Arg404 were replaced by alanine and a significant ∼100-fold increase in *K*_*d*_ observed for Asn134, highlighting that these residues are essential for substrate recognition and binding. In contrast, binding of trehalose was still observed when Asn25, Glu26, Gln59, and Leu335 were replaced by an alanine, with a corresponding ∼3-fold reduction in *K*_*d*_ for the Asn25 and Glu26 mutants and ∼10- and ∼13-fold reduction in the *K*_*d*_ values for Gln59 and Leu335, respectively, compared with wild-type *Mtr* LpqY. This indicates that while these amino acids are important for binding, mutations within these regions can be tolerated and are less critical for trehalose recognition. Examination of the sequence alignments reveals that the Asp25 and Glu26 residues are not conserved between mycobacterial homologs and that an alanine residue at position 25 naturally occurs in *Mycobacterium marinum* LpqY (Fig. S8). In contrast, the *Mtr* LpqY Asn25-Glu26 double mutant that mimics the *Mtb* LpqY binding site resulted in a higher binding affinity for trehalose and has the same substrate profile as *Mtr* LpqY (Fig. S10) indicating that these nonconserved residues have an important role in the recognition of the trehalose substrate in *Mtb*.

**Table 2:** Binding data for *Mtr* LpqY mutants

*Mtr* LpqY	*K*_*d*_ (μM)
WT	72.1 ± 3.1
Asn25Ala	206.5 ± 4.0
Asn25Thr-Glu26Asp	23.4 ± 0.5
Glu26Ala	233.9 ± 9.3
Gln59Ala	690.8 ± 19.2
Asp80Ala^a^	---
Asn134Ala	9061 ± 11.7
Glu241Ala	NBD
Trp259Ala	NBD
Leu335Ala	945.7 ± 46.6
Arg404Ala	NBD

*Mtr*, *Mycobacterium thermoresistible*; NBD, No binding detected.

a “---” The *K*_*d*_ for Asp80Ala mutant was too unstable to label and could not be determined. Mean ± SEM are from at least three independent experiments.

### Molecular dynamics simulations of Mtr LpqY

**Figure 4:**
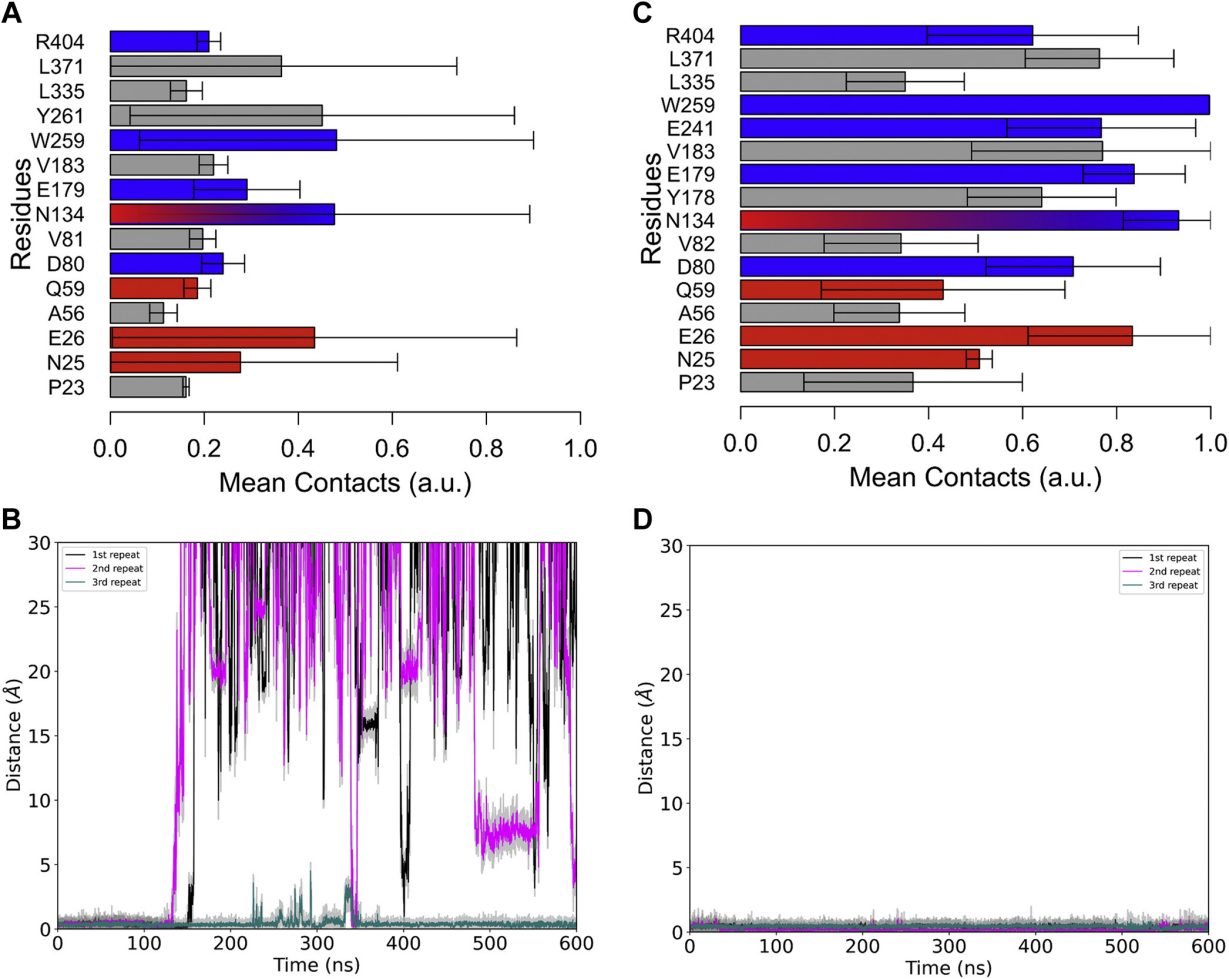
**Molecular dynamic simulations of *Mtr* LpqY.***A* and *B*, Glu256 not protonated; *C* and *D*, Glu 256 protonated. *A and C*, Residues of *Mtr* LpqY interacting with trehalose over the course of the simulation, where 1 is in contact for the entire simulation. Data from three repeats of 600 ns are shown, where the error bars represent standard deviation. *Blue bars* signify hydrogen bonds with Glc-1, *red bars* signify hydrogen bonds with Glc-2, and *gray bars* show hydrophobic contacts. *B* and *D*, The minimum distance of trehalose from W259 (roughly center of the binding site) over the course of a simulation. Data from three repeats of 600 ns are shown, and the colored line represents the local running average of the associated repeat with the *gray line* showing data points of that simulation. *Mtr*, *Mycobacterium thermoresistible*.

To further explore the interactions between trehalose and *Mtr* LpqY, molecular dynamic (MD) simulations were performed over three repeats of 600 ns (Fig. 4, Movies S1 and S2). The simulations identified that trehalose has an unexpectedly short retention time in the binding pocket of ∼130 to 150 ns (Fig. 4*B*). Upon release of the sugar, *Mtr* LpqY undergoes a closed-to-open transition with a 131° rotation opening of the two domains, calculated from DynDom (25) (Fig. S11), typical of the “Venus-fly trap mechanism” reported for other substrate binding proteins (23). As the initial set of simulations were performed with amino acids set at their default protonation states, we analyzed whether any of the side chains had a predicted nonstandard pK_a_ value, based on the coordinates of the crystal structure, using the PROPKA tool (26). This identified Glu256, located on β9, to be of interest as it was found to have a high pK_a_ of 8.4 in the crystal structure, compared with an expected value of 4.5, which suggests in this conformation it could be protonated. The simulations were therefore repeated with Glu256 protonated and compared with the results of the deprotonated form. Unlike the previous simulations, trehalose remained within the *Mtr* LpqY binding site for the entirety of each repeat, despite Glu256 being distant from the trehalose binding site. Comparison between the sets of simulations can be seen in Figure 4, with contacts between *Mtr* LpqY and trehalose agreeing with those observed in the X-ray structure (Figs. 3 and 4). The residues that were identified to be critical for trehalose binding, Asn134, Glu241, Trp259, and Arg404 (Table 2), maintained contact with the disaccharide for the majority of the simulation, further highlighting their importance in sugar recognition. A notable difference between the protonated and deprotonated simulations is an increased interaction with Glu241 when Glu256 is protonated (Fig. 4*C*). Analysis of our structure identified that Glu256 may influence the interaction of Glu241 with trehalose *via* a hydrogen bond bridging interaction with Asn258. Indeed, our simulation data indicate that protonation of Glu256 results in an increased contact of Asn258 with Glu241. We postulate that the increased hydrogen bonding availability of Asn258 stabilizes the interaction of LpqY with trehalose (Fig. S12). Overall, our results suggest that the contacts between Glu241 and trehalose could be significant in retaining the disaccharide until LpqY engages with the SugABC transporter.

### Saturation transfer difference NMR of Mtr LpqY with trehalose and 6-azido-trehalose

**Figure 5:**
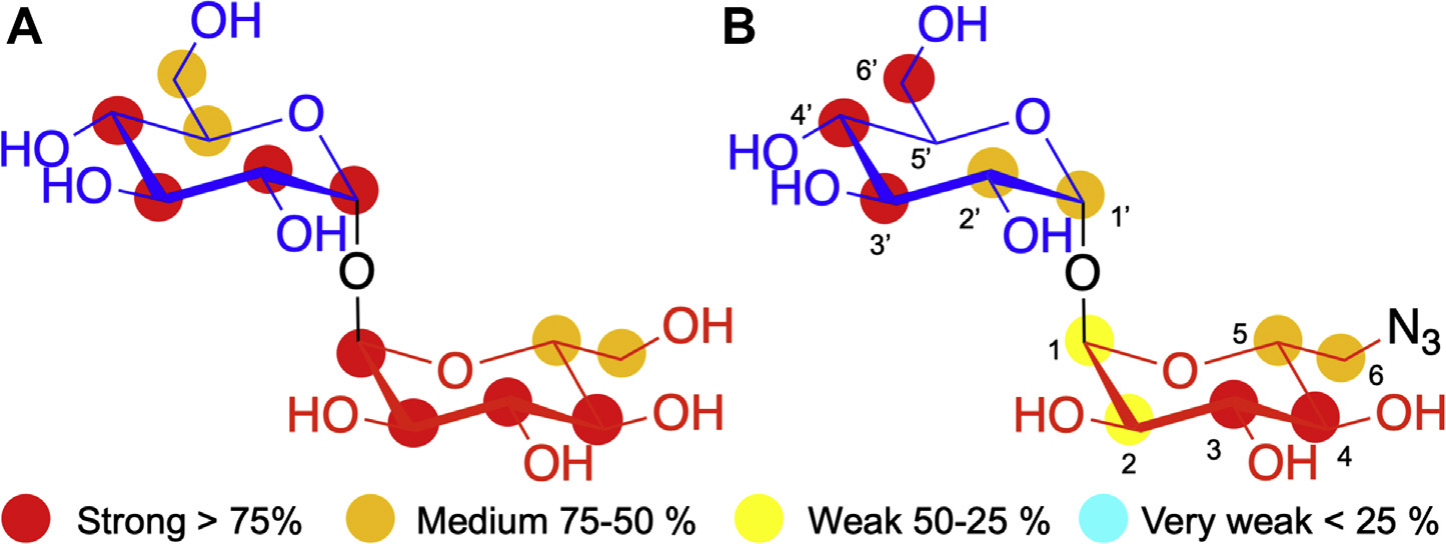
**STD NMR binding of trehalose and 6-azido-trehalose to *Mtr* LpqY**. *A*, binding epitope map of trehalose and (*B*) 6-azido-trehalose. Protein saturation was achieved by irradiation at 0.80 ppm. The colored spheres represent normalized STD NMR intensities. STD responses are only indicated for protons that could accurately be measured. Carbon atom nomenclature is indicated for 6-azido-trehalose. Glc-1 (*blue*), Glc-2 (*red*). *Mtr*, *Mycobacterium thermoresistible*; STD NMR, saturation transfer difference NMR.

Azide-modified trehalose analogs coupled with biorthogonal “click” labeling are useful tools to investigate trehalose uptake and metabolism in mycobacteria (13). However, despite numerous efforts, we were unable to obtain a crystal structure of *Mtr* LpqY in complex with 6-azido-trehalose. Therefore, to further our understanding into the mode of ligand binding, the binding epitope of 6-azido-trehalose with *Mtr* LpqY was determined in solution by saturation transfer difference (STD) NMR experiments, as described in the Methods section. The binding of trehalose to *Mtr* LpqY was also assessed by STD NMR to establish its binding epitope in solution and enable comparison with our X-ray structure. Binding was confirmed for both trehalose and 6-azido-trehalose, and the corresponding epitope maps are shown in Figure 5. For both ligands, STD NMR signals were obtained for each hydrogen atom from both glucose units (Fig. S13), indicating that both carbohydrate rings are important in binding recognition. As a result of the C2 symmetry of the trehalose disaccharide, identical binding epitopes were obtained for each glucose unit (Fig. 5 and Fig. S13). Strong STD intensities for protons in positions 1 to 4 were observed suggesting that these are in close contact with *Mtr* LpqY, whereas medium intensity values were observed for protons in positions 5 and 6 (Fig. 5). In direct contrast, a different STD NMR intensity pattern was determined for the unsymmetrized 6-azido-trehalose derivative (Fig. 5*B*) indicating that the azido-modified analog binds in a single orientation within the *Mtr* LpqY binding pocket. Notably, the 6-azido modified glucose ring displays an overall decrease in the relative STD intensities. In particular, weak STD NMR signals for protons in positions 1 and 2 are observed, which is compatible with the lower binding affinity observed for the azide-modified analog (Table 1).

**Figure 6:**
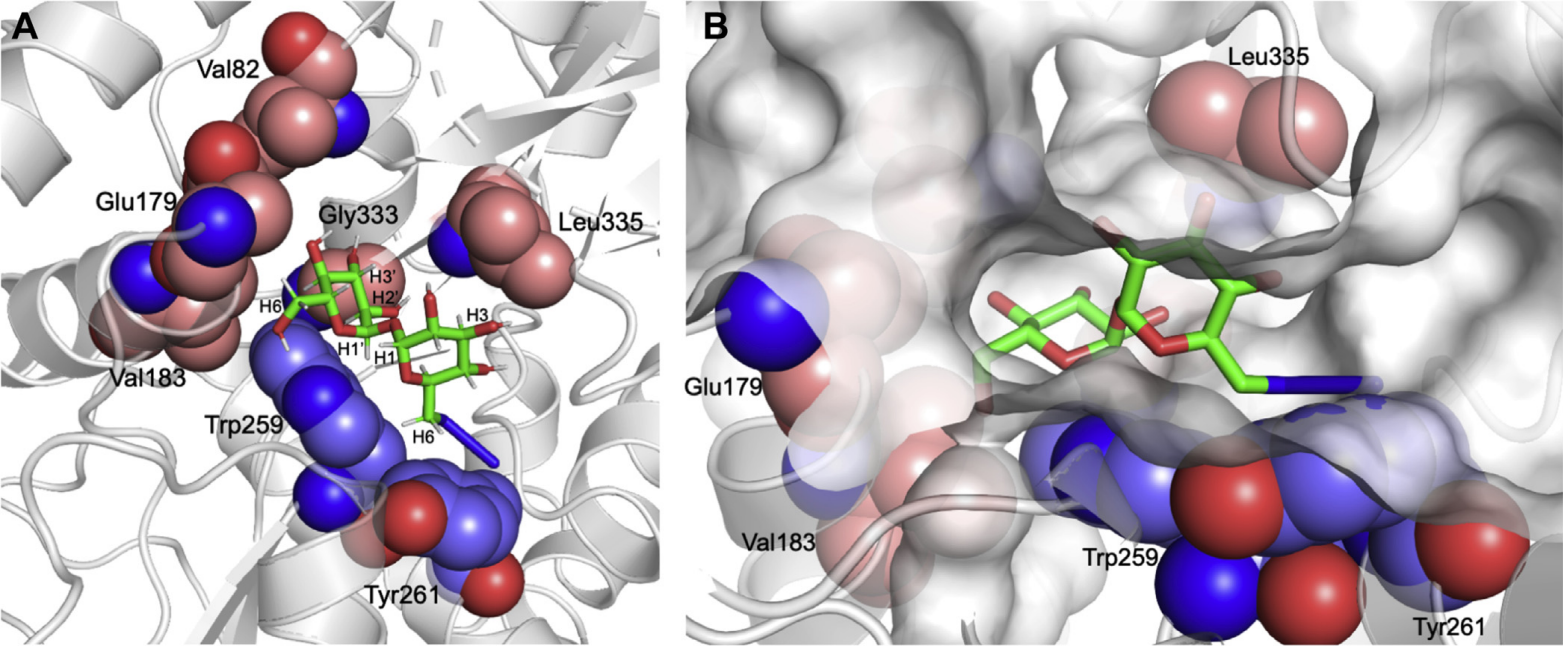
**6-azido-trehalose binding to *Mtr* LpqY.** Model of the mode of binding of 6-azido-trehalose to *Mtr* LpqY based on experimental DEEP-STD NMR derived interactions (see Fig. S14*B*). *A*, hydrogens showing significant DEEP-STD factors with aliphatic (*red carbon spheres*) and aromatic residues (*blue carbon spheres*). *B*, surface representation of *Mtr* LpqY. Trehalose is shown in stick format: carbon atoms in *green*, oxygen atoms in *red*, nitrogen atoms in *blue*, hydrogen atoms in *gray*. DEEP-STD NMR, differential epitope mapping saturation transfer difference NMR; *Mtr*, *Mycobacterium thermoresistible*.

To probe for additional structural information in the solution state and gain information about the orientation of the ligand within the binding site and the type of amino acids contacting the hydrogen atoms of the bound ligand, we then utilized the recently developed differential epitope mapping STD NMR (DEEP-STD NMR) approach (Fig. S14) (27). This has been successfully applied to study other ABC transporters in gut bacteria (28). The DEEP-STD maps highlight differences in the orientations of ligand protons related to protein aliphatic and aromatic side chains in the binding pocket and clearly indicated that the molecular determinants of trehalose binding to *Mtr* LpqY correlate in both solution and solid states. In the case of 6-azido-trehalose, individual DEEP-STD intensity patterns for each monosaccharide were observed indicating that protons from both glucose rings make distinct close contacts to *Mtr* LpqY (Fig. S14*B*). Specifically, the H1, H1’ H2, H6a, and H6b protons are orientated toward aromatic residues, and the H3, H2’, H3’, and H6’ are orientated toward aliphatic side chains (Fig. S14). Given the possibility that the azide-containing glucose ring could bind in either glucose subsite, we modeled the binding of 6-azido-trehalose based on the experimental NMR-derived interactions (Fig. 6). Altogether, these results indicate that the unmodified glucose ring is positioned at the base of the *Mtr* LpqY binding pocket, with the 6-azido-glucose ring accommodated at the second subsite located toward the channel entrance with the 6-azido-group extending into an expanded binding pocket in this region (Fig. 6*B*).

## Discussion

The ongoing battle of *Mtb* to assimilate scarce nutrients during intracellular infection is a critical factor for the survival of this major global pathogen. Trehalose is a key component of the mycobacterial cell envelope, and “free” trehalose, released from the trehalose-containing glycolipids, is recovered by the LpqY-SugABC ABC transporter (6). Significantly, a functioning trehalose transport system is essential for *Mtb* to establish infection (6) and has no obvious human homolog, and for these reasons, this importer has been implicated as a target for the development of new antitubercular agents and diagnostic tools.

We sought to investigate the substrate specificity and molecular basis of trehalose recognition of the mycobacterial LpqY substrate binding protein. Altogether, our results provide a number of important new insights. Significantly, our biochemical, X-ray crystallographic, MD simulation, and STD NMR data are consistent and provide the first direct evidence that *Mtr* LpqY is highly specific for trehalose. It is particularly noteworthy that the *Mtr* LpqY-SugABC transporter does not recognize alternative monosaccharides or disaccharides or known substrates of other *Mtb* carbohydrate importers which further underscores the notion that each *Mtb* carbohydrate importer has a distinct substrate preference (18, 19). Further experiments are now underway to link the recognition of carbohydrates by LpqY with uptake by the LpqY-SugABC transport system.

Our *Mtr* LpqY co-complex crystal structure in combination with STD NMR provides a unique insight into the molecular basis of trehalose recognition and substrate specificity in *Mtb*. Notably, trehalose specificity is manifested through a network of hydrogen bond interactions which link each hydroxy group from both glucose moieties to residues located within the LpqY binding pocket. These interactions have a pronounced effect on substrate recognition and dictate the stringent stereoselective requirement for an α1,1 linked disaccharide. It is particularly interesting to highlight the inability of *Mtr* LpqY to bind maltose (α1-4) as this feature differs significantly from α-glycoside disaccharide transporters from *Thermus* sp. that bind multiple carbohydrates, including glucose (17, 29). Consistent with the low sequence identity between these substrate-binding proteins (PDB 6J9W: 23%, PDB 1EU8: 27.5%), there are significant differences between the carbohydrate binding motifs of these organisms that originate from different regions of the proteins (Figs. S15 and S16). We propose that *Mtb* has evolved unique structural features to facilitate the specific import of the main disaccharide present in its niche host environment compared with the diversity of sugars available in geothermal habitats. As expected, given the lack of genes encoding for phosphotransferase systems in *Mtb*, *Mtr* LpqY did not recognize trehalose-6-phosphate. Modified trehalose derivatives have been developed as tools to probe trehalose processing pathways in mycobacteria; however, up until now, the structural basis for the selective recognition of these analogs was unknown (11, 12, 13). Notably, our STD NMR studies in combination with MST analyses support the uptake of the 6-azido-trehalose analog by the mycobacterial LpqY-SugABC transporter and explain the reduced affinity for this chemically modified substrate. It is particularly interesting to note that through the systematic evaluation of substrate specificity, we observe that *Mtr* LpqY is promiscuous for alternative trehalose derivatives modified at each position. Previous studies have shown that while 2-, 4-, and 6-azido trehalose analogs are imported and found in the cytosol, 3-azido-trehalose is not (13). Interestingly, our binding studies indicate that *Mtr* LpqY has similar affinity for 3- and 6-azido-trehalose suggesting that while 3-azido-trehalose binds to *Mtr* LpqY, it is a noncognate ligand and is not transported by LpqY-SugABC. This finding may have interesting implications in the design of inhibitors of this essential ABC transporter.

Our understanding of how the LpqY-SugABC transporter ensures an efficient intracellular supply of trehalose to mycobacteria is still evolving. However, our structural and MD simulations suggest an important role in the protonation state of Glu256. It is likely that the protonation of the Glu256 side chain stabilizes the interaction of trehalose in the binding pocket. This is supported by the observation that when Glu256 is protonated, trehalose remains within the *Mtr* LpqY binding pocket for the entire simulation. Analysis of the contacts suggests that a significant contribution to sugar recognition is from Glu241, which is mediated through Asn258. The observation that the protonation state of a side chain within a substrate binding protein influences the stability of substrate recognition raises new questions into the mechanistic basis of transport of ABC transporters.

In conclusion, one of the major hurdles in TB drug development is that molecules need to penetrate the mycobacterial cell envelope to gain intracellular access and kill *Mtb*. However, the complex *Mtb* cell envelope poses a significant impermeable barrier, which prevents drugs and diagnostic tools from accessing the cytoplasm. The opportunities of targeting the vulnerable *Mtb* LpqY-SugABC transporter are two-fold. First, the extracellular location of the LpqY substrate binding lipoprotein component provides a route to develop TB drugs that can kill *Mtb* without needing to cross the impermeable cell envelope. Second, it offers the opportunity to hijack this import system to deliver potent inhibitor substrate mimics into the mycobacterial cell. The results from this work represent a significant step in this direction and provide a robust framework to ultimately exploit this transporter in the rational development of new antitubercular agents and diagnostic tools.

## Experimental procedures

All chemicals and reagents were purchased from Sigma-Aldrich or Carbosynth, unless specified. PCR and restriction enzymes were obtained from New England Biolabs. Double-distilled water was used throughout.

### Plasmid construction

*M. thermoresistible* (NCTC 10409) was obtained from the Public Health England National Collection of Type cultures, and gDNA was isolated using established protocols (30). The *lpqY* gene was amplified from *Mtr* genomic DNA by PCR using gene-specific primers (Table S2) based on the annotated sequence retrieved from the NCBI database (GenBank LT906483). It is possible that the start codon for the *Mtr lpqY* gene starts further upstream than that annotated in the NCBI database, in which case the *Mtr* LpqY protein is a truncated version. The PCR amplification (Q5 polymerase [NEB]) consisted of 30 cycles (95 °C, 2 min; 95 °C, 1 min; 60 °C, 30 s; 72 °C, 3 min), followed by an extension cycle (10 min at 72 °C). The resulting PCR product was cloned into a modified pET-SUMO vector (a gift from Dr Patrick Moynihan, University of Birmingham) using the *BamHI* and *HindIII* restriction enzyme sites resulting in the construct *mtr_lpqY_sumo*. Targeted single-site substitutions were introduced into *mtr_lpqY_sumo* using the primers that are detailed in Table S2, with Phusion HF polymerase, and the PCR cycle (98 °C, 30 s; 20 cycles of 98 °C, 30 s; 60 °C, 30 s; 72 °C, 4 min; followed by 5 min at 72 °C), followed by digestion with 1 μl DpnI. All plasmid sequences were verified by DNA sequencing (GATC) and used for protein expression.

### Recombinant overexpression of Mtr LpqY

*E. coli* BL21 (DE3) competent cells were transformed with the appropriate *mtr*_*lpqY_sumo* expression plasmid and grown at 27 °C to an optical density at 600 nm (OD_600_) of 0.6 to 0.8 in terrific broth medium supplemented with 50 μg/ml kanamycin. Protein production was induced with 1 mM isopropyl-β-thiogalactopyranoside, and the cultures were grown at 16 °C overnight with shaking (180 rpm). The cells were harvested (4000 *g*, 30 min, 4 °C) and resuspended in lysis buffer (20 mM Tris, 300 mM NaCl, 10% glycerol, pH 7.5 [buffer A]) supplemented with 0.1% Triton X-100 and frozen at −80 °C until further use.

### Protein purification

A complete protease inhibitor tablet (Roche), 5 mM MgCl_2_, 2 mg of DNase, and 20 mg of lysozyme were added to the resuspended pellet, and the pellet was sonicated on ice (Sonicator Ultrasonic Liquid Processor XL; Misonix). Following centrifugation (39,000*g*, 30 min, 4 °C), the supernatant was filtered (0.45 μm filter) and loaded onto a pre-equilibrated HisPur Ni^2^-NTA affinity resin (Thermo Scientific). The column was washed with buffer A (5 column volumes), and the recombinant *Mtr* LpqY protein was eluted from the Ni^2+^ resin with increasing concentrations of imidazole. Fractions containing the *Mtr* LpqY protein were digested with His-tagged SUMO protease (1 h, 30 °C, 300 μg) and dialyzed at 4 °C for 12 h against buffer A. A second HisPur Ni^2^-NTA affinity resin purification step was undertaken, and the fractions containing *Mtr* LpqY protein were pooled and purified further using size exclusion chromatography (Superdex 200 16/600 column, GE Healthcare) with buffer A. Fractions containing *Mtr* LpqY were combined, and a final HisPur Ni^2^-NTA affinity resin purification step was undertaken with buffer A. The flow-through fractions containing purified *Mtr* LpqY were pooled, and the protein was concentrated to 5 to 14 mg/ml (Vivaspin 20; GE Healthcare) and stored at −80 °C. The identity of the proteins were confirmed by tryptic digest and nanoLC–electrospray ionization–MS/MS (WPH Proteomics Facility, University of Warwick).

### Circular dichroism analysis

Purified *Mtr* LpqY proteins were diluted to 0.2 mg/ml and dialyzed in the following buffer: 20 mM Tris, 20 mM NaCl, pH 7.5. The samples were transferred into a 1 mm path length quartz cuvette and analyzed on Jasco J-1500 DC spectrometer from 198 to 260 nm. Spectra were acquired in triplicate and averaged after subtraction of the buffer background.

### Crystallization and structure determination

For co-crystallization experiments, *Mtr* LpqY was buffer exchanged into 20 mM HEPES, 20 mM NaCl pH 7.5 and incubated with 10 mM trehalose at room temperature for 10 min. Successful crystallization required the removal of unbound trehalose through a series of concentration and dilution wash-steps (Vivaspin 20; GE Healthcare) before crystallization. Crystals of *Mtr* LpqY in complex with trehalose were grown by vapor diffusion in 96-well plates (Swiss-Ci) using a Mosquito liquid handling system (TTP LabTech) by mixing 1:1 volumes (100 nl) of concentrated LpqY (14 mg/ml) with reservoir solution. *Mtr* LpqY crystals typically grew within 3 to 7 days at 22 °C in 0.1 M HEPES pH 6.0, 50% w/v polypropylene glycol 400, 5% DMSO, and 1 mM TCEP. The *Mtr* LpqY crystals were either directly flash-frozen in liquid nitrogen before data collection or soaked in 1 M NaI prepared in the same crystallization buffer for 5 min before freezing. The X-ray diffraction data for the ligand bound *Mtr* LpqY crystals and iodide derivatives were collected at the I03 beamline of Diamond Light Source. All diffraction data were indexed, integrated, and scaled with XDS (http://xds.mpimf-heidelberg.mpg.de/) (31) through the XIA2 pipeline and the CCP4 suite of programs (32). Initial phases were determined based on an iodide derivative through the Big_ep phasing pipeline (33). An initial model of *Mtr* LpqY was generated using Autobuild (34). This structural model was used to determine a molecular replacement solution (Phaser (35)) for a native *Mtr* LpqY data set, and refinement was carried out in phenix-refine (36) and manual rebuilding in COOT (37). The find ligand function in COOT was used to fit the trehalose ligand into unoccupied electron density in both chains of the asymmetric unit. The restraints for use in refinement were calculated using REEL (38). The model of the ligand-bound structure comprises residues 14 to 448 in both chains (A-B). No Ramachandran outliers were identified, and structure validations were done by MolProbity (39). Figures were prepared using Pymol (The PyMOL Molecular Graphics System, Version 2.0 Schrödinger, LLC), except for those showing electron density which were prepared using CCP4mg (40).

### ^1^H STD NMR experiments

All the STD NMR experiments were performed in PBS D_2_O buffer, pH 7.4. For the LpqY/trehalose complex, the protein concentration was 25 μM, whereas the ligand concentration (trehalose or 6’-azido-6’-deoxy-trehalose) was 1 mM. STD NMR spectra were acquired on a Bruker Avance 500.13 MHz at 288 K. The on- and off-resonance spectra were acquired using a train of 50 ms Gaussian selective saturation pulses using a variable saturation time from 0.5 s to 5 s, and a relaxation delay (D1) of 4 s. The water signal was suppressed using the watergate technique (41), whereas the residual protein resonances were filtered using a T_1ρ_-filter of 40 ms. All the spectra were acquired with a spectral width of 8 kHz and 24K data points using 512 scans. The on-resonance spectra were acquired by saturating at 0.80 (aliphatic hydrogens) or 7.20 ppm (aromatic hydrogens), as average chemical shifts predicted from shiftX2 (42) for the aliphatic and aromatic residues present in the binding site of *Mtr* LpqY, whereas the off-resonance spectra were acquired by saturating at 40 ppm. To get accurate structural information from the STD NMR data and to minimize the T_1_ relaxation bias, the STD build up curves were fitted to the equation STD(t_sat_) = STD_max_∗(1-exp(-k_sat_∗t_sat_)), calculating the initial growth rate STD_0_ factor as STD_max_∗k_sat_ = STD_0_ and then normalizing all of them to the highest value (43). DEEP-STD factors were obtained as previously described (27) after a saturation time of 1 s on aliphatic or aromatic regions (0.80 or 7.20 ppm, respectively).

### Docking calculations

Schrodinger’s Maestro 2019 to 1 suite was used to dock both disaccharides into *Mtr* LpqY, employing the crystal structure of *Mtr* LpqY in complex with trehalose. First, the water molecules and ions were removed using the Protein Preparation Wizard tool, and the protonation state for each residue was calculated with Epik at pH 7.5. Both ligands (trehalose and 6’-azido-6’-deoxy-trehalose) were prepared using Ligprep. Before the docking calculation, a receptor grid was generated with Glide setting a square box centered on the trehalose in the crystal structure (then removed) of 20 Å side. Trehalose and 6’-azido-6’-deoxy-trehalose were then docked with Glide with extra precision, and a postdock minimization was performed. Data were processed, and figures prepared with the Maestro suite.

### Thermal shift assay

The transition unfolding temperature *T*_m_ of the *Mtr* LpqY protein (2.6 μM) was determined in the presence or the absence of ligands. The screen used a final ligand concentration of 10 mM. Reactions were performed in a total volume of 20 μl using Rotor-Gene Q Detection System (Qiagen), setting the excitation wavelength to 470 nm and detecting emission at 555 nm of the SYPRO Orange protein gel stain, 31 × final concentration (Invitrogen, 5000X concentrate stock). The cycle used was a melt ramp from 30 to 95 °C, increasing temperature in 1 °C steps and time intervals of 5 s. Fluorescence intensity was plotted as a function of temperature. The *T*_m_ was determined using the Rotor-Gene Q software and the Analysis Melt functionality. All experiments were performed in triplicate.

### Isothermal titration calorimetry

ITC experiments were performed using the PEAQ-ITC system (Malvern Panalytical Ltd) at 25 °C. *Mtr* LpqY was dialyzed extensively into 50 mM HEPES, 300 mM NaCl, pH 7.5, and the trehalose and galactotrehalose ligands was dissolved in this dialysis buffer. The syringe was loaded with the ligand (500 μM), and the calorimetric cell was loaded with *Mtr* LpqY (53.6 μM). Following a 60 s initial equilibration, an initial injection of 0.4 μl was performed followed by 19 injections of 2.0 μl every 120 s with a speed of injection of 0.5 μl/s. The data were analyzed using the “one set of sites” model within the MicroCal PEAQ-ITC software (Malvern) iterated using the Lavenberg-Marquardt algorithm after subtraction of the control experiment (trehalose titrated into buffer). The thermodynamic and binding parameters were derived from the nonlinear least squares fit to the binding isotherm.

### Microscale thermophoresis

The *Mtr* LpqY protein was labeled using the amine reactive RED-NHS dye (3 μM) (second generation, NanoTemper Technologies) and a constant concentration of *Mtr* LpqY (2.6 μM). Excess dye was removed by size exclusion chromatography (Superdex 200 10/300 column [GE Healthcare] using 50 mM HEPES, 300 mM NaCl, pH 7.5). The compounds were prepared in PBS containing 0.05% Tween 20, and the final concentration of the protein in the assay was 500 nM. The samples were loaded into the MonoLith NT.115 standard treated capillaries and incubated for 10 min before analysis using the Monolith NT.115 instrument (NanoTemper Technologies) at 21 °C using the auto-select excitation power (20%) and medium laser power. The binding affinities were calculated using a single-site binding model using the MST NT Analysis software (version 7.0). All experiments were carried out in triplicate.

### Atomistic simulations

All simulations were run using GROMACS 2019 (44). Simulations of the LpqY X-ray structure were performed without position restraints for a total of 600 ns and run in triplicate. In all cases, a 2 fs timestep was used, in an NPT ensemble with V-rescale temperature coupling at 310 K (45) and a semi-isotropic Parrinello-Rahman barostat at 1 bar, with protein/trehalose and water/ions coupled individually (46). Electrostatics were described using the Particle Mesh Ewald method, with a cut-off of 1.2 nm, and the van der Waals interactions were shifted between 1 and 1.2 nm. The tip3p water model was used. The water bond angles and distances were constrained by SETTLE (47). Hydrogen covalent bonds were constrained using the LINCS algorithm (48). Analysis was performed using MDAnalysis (49) and visualized in PyMOL. Protonation state calculations were performed using PROPKA3 (26).

### Synthesis

A full description of all methods for the synthesis and characterization of all compounds are provided in the supporting information.

## Data availability

The structure presented in this article has been deposited in the Protein Data Bank (PDB) with the following code: 7APE. All remaining data are contained within the article.

## Conflict of interest

The authors declare that they have no conflicts of interest with the contents of this article.
