# Supplementary material for: Structural basis of trehalose recognition by the mycobacterial LpqY-SugABC transporter
Source: J Biol Chem. 2021 Jan 19;296:100307. doi: 10.1016/j.jbc.2021.100307 (PMC7949145; doi:10.1016/j.jbc.2021.100307)
Supplement: Supplemental Figure S1–S16 and Tables S1–S2 [file mmc3.pdf]

Supporting information

Structural basis of trehalose recognition by the mycobacterial LpqY-SugABC transporter

Christopher M. Furze[1], Ignacio Delso[2,3], Enriqueta Casal[3], Collette S. Guy[1], Chloe Seddon[1], Chelsea M. Brown[1], Hadyn L. Parker[1], Anjana Radhakrishnan[1], Raul Pacheco-Gomez[4], Phillip J. Stansfeld[1,5], Jesus Angulo[3,6,7], Alexander D. Cameron[1], Elizabeth Fullam[1]*

[1]School of Life Sciences, University of Warwick, Coventry, CV4 7AL, UK
[2] Instituto de Síntesis Química y Catálisis Homogénea (ISQCH), Universidad de Zaragoza, CSIC, 50009 Zaragoza, Spain
[3] School of Pharmacy, University of East Anglia, Norwich Research Park, Norwich, Norfolk NR4 7TJ, UK
[4] Malvern Panalytical Ltd, Enigma Business Park, Grovewood Road, Malvern, WR14 1XZ, United Kingdom
[5] Department of Chemistry, University of Warwick, Coventry, CV4 7AL, UK
[6] Departamento de Química Orgánica, Universidad de Sevilla, C/ Prof. García González, 1, 41012 Sevilla, Spain.
[7] Instituto de Investigaciones Químicas (CSIC-US), Avda. Américo Vespucio, 49, 41092 Sevilla, Spain.

*To whom correspondence should be addressed: Elizabeth Fullam, School of Life Sciences, University of Warwick, Coventry, CV4 7AL, United Kingdom; e.fullam@warwick.ac.uk; Tel. +44 (0)2476 574239

Running title: Structure/function of *Mtr*-LpqY

Table of Contents

Supplementary Results

Fig. S1. Sequence alignment of LpqY from *Mycobacterium thermoresistible* with LpqY from *Mycobacterium tuberculosis* 3
Fig. S2. SDS-PAGE analysis of the purification of *Mycobacterium thermoresistible* LpqY 4
Fig S3. Thermal shift assay probing a panel of potential LpqY ligands 5
Fig. S4. Structure of selected carbohydrates probed in the thermal shift assay 6
Fig. S5. ITC of *Mtr* LpqY 7
Fig. S6: MST of *Mtr* LpqY and trehalose 8
Fig S7. Electron density for the trehalose substrate. 9
Fig. S8. Sequence alignment of mycobacterial LpqY homologues 10
Fig S9. Circular dichroism spectra of *Mtr* LpqY and site directed mutant proteins. 11
Fig. S10. Thermal shift assay against the *Mtr* LpqY Asn25Thr-Glu26Asp double mutant 12
Fig. S11. Post 600 ns simulation snapshot of *Mtr* LpqY 13
Fig. S12. Interactions of *Mtr* LpqY Asn258 14
Fig. S13. STD NMR for *Mtr* LpqY with trehalose and 6-azido-trehalose 15
Fig. S14. Differential Epitope Mapping by STD NMR of *Mtr* LpqY with trehalose and 6-azido-trehalose 16
Fig. S15. Comparison of *Mtr* LpqY with *Thermus sp.* 17
Fig. S16. Sequence alignment of *Mtr* LpqY with *Thermus sp.* homologues 18
Table S1. Data collection and statistics for *Mtr* LpqY in complex with trehalose 19
Table S2. Sequence of primers for cloning and site-directed mutagenesis 20

Materials and Methods

Synthetic Methods 21
^{2}H-trehalose 21
6-bromo-6-deoxy-α,α′-trehalose 21
6-azido-6-deoxy-α,α′-trehalose 22
6-amino-6-deoxy-α,α′-trehalose 22
2,3,6,2′,3′,4′,6’,-hepta-*O*-benzoyl-α,α-D-trehalose 22
2,3,6,-tri-*O*-benzoyl-α-D-galactopyranosyl-(1→1)-2′,3′,4′,6′,-tetra-*O*-benzoyl-α-D-glucopyranoside 23
4-Azido-2,3,6,-tri-*O*-benzoyl-α-D-galactopyranosyl-(1→1)-2′,3′,4′,6′,-tetra-*O*-benzoyl-α-D-glucopyranoside 23
4-Azido-4-deoxy-α,α′-trehalose 24
α-D-galactopyranosyl-(1→1)-α-D-glucopyranoside: galactotrehalose 24
Chemoenzymatic synthesis of α-D-mannopyranosyl-(1→1)-α-D-glucopyranoside: mannotrehalose 24
Chemoenzymatic synthesis of 3-azido-trehalose 25
Chemoenzymatic synthesis of *N*-acetyl-2-amino-2-deoxy- α,α′-trehalose 25
2-azido-trehalose 26

References

References 27

Fig. S1. Sequence alignment of LpqY from *Mycobacterium thermoresistible* with LpqY from *Mycobacterium tuberculosis*. The sequence alignment was generated using Clustal Omega (1) and ESPript version 3.0 (2). Identical residues are indicated by a red background, conserved residues by red characters and similar residues outlined in a blue box. Blue stars indicate *Mtr* LpqY residues that interact with trehalose.

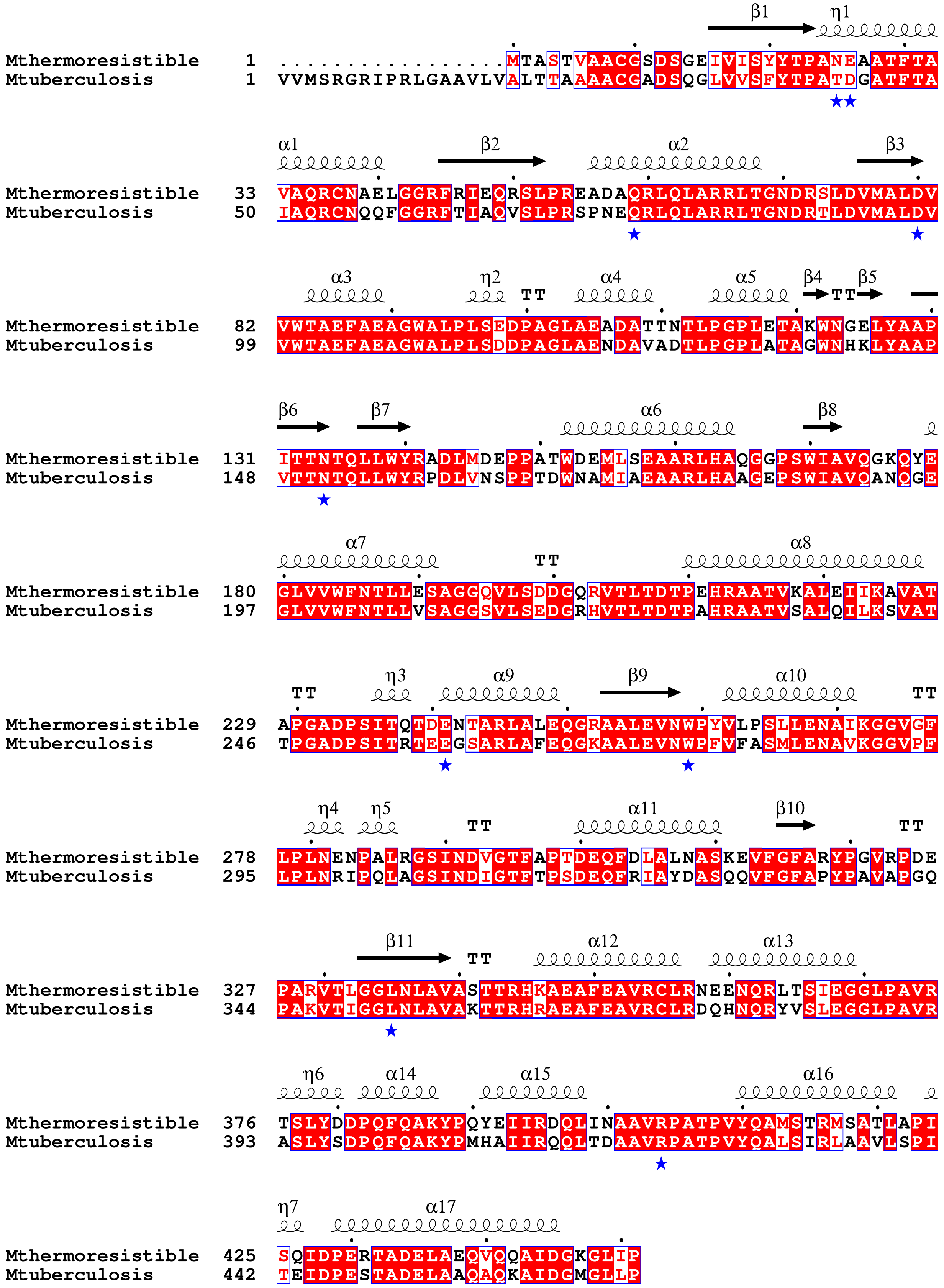

Fig. S2. SDS-PAGE analysis of the purification of *Mycobacterium thermoresistible* LpqY.

A) Elution of Sumo-His$_6$-tagged *Mtr* LpqY from a Ni^{2+} IMAC-column. M = molecular weight marker in kDa, WC = whole cell, S = soluble lysate, IS = insoluble fraction, FT = flow through, W= buffer wash (0 mM imidazole), numbers 5 – 1000 refer to the imidazole concentration in the elution buffer (units of mM)

B) Ni^{2+} IMAC-column of *Mtr* LpqY following digestion of the Sumo-His$_6$ tag with SUMO protease. L = undigested LpqY, D = digested LpqY, FT = flow through, W1 = first wash, W2 = second wash, W3 = third wash, 250-1000 refer to the imidazole concentration in the elution buffer (units of mM).

C) Gel filtration chromatography of *Mtr* LpqY following digestion and removal of the Sumo-His$_6$ tag with the volumes shown corresponding to Fig. S2D**.**

D) Gel filtration trace with absorbance measured at 280 nm.

E) Ni^{2+} IMAC-column of *Mtr* LpqY following gel filtration. GF = pooled gel filtration fractions, FT = flow through, W = wash, IMZ = 1 M imidazole elution. See Methods for buffer compositions.

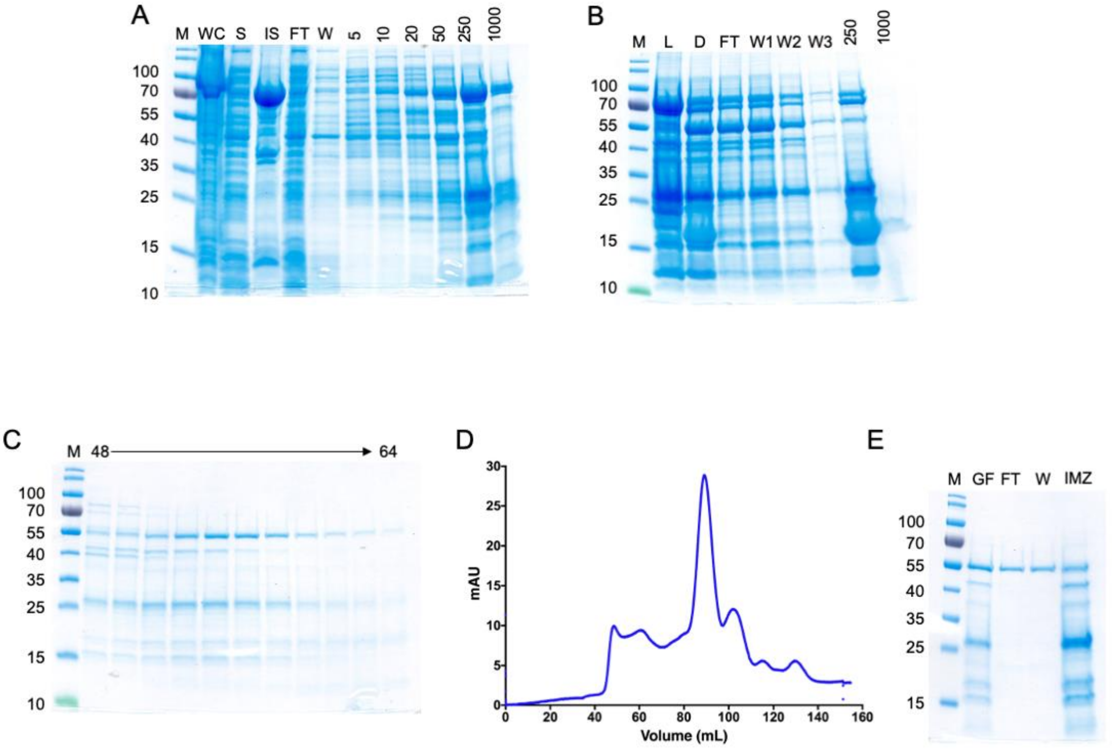

Fig. S3. Thermal shift assay probing a panel of potential LpqY ligands. Bar graphs illustrating the ΔT_m shifts for the series of potential ligands probed for binding at a final concentration of 10 mM. Data are shown from three independent repeats represented as mean ± SD.

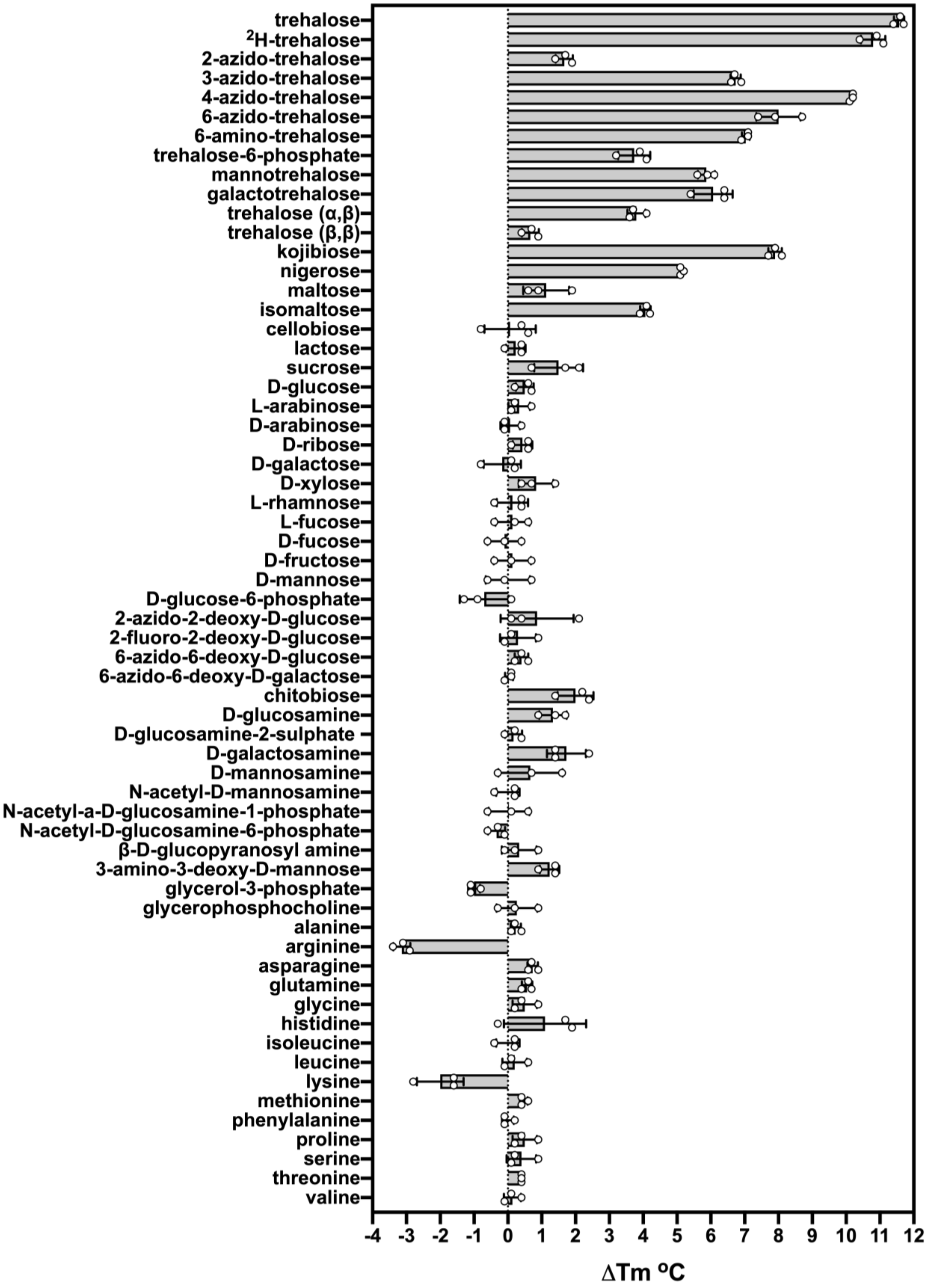

Fig. S4. Structure of selected carbohydrates probed in the thermal shift assay

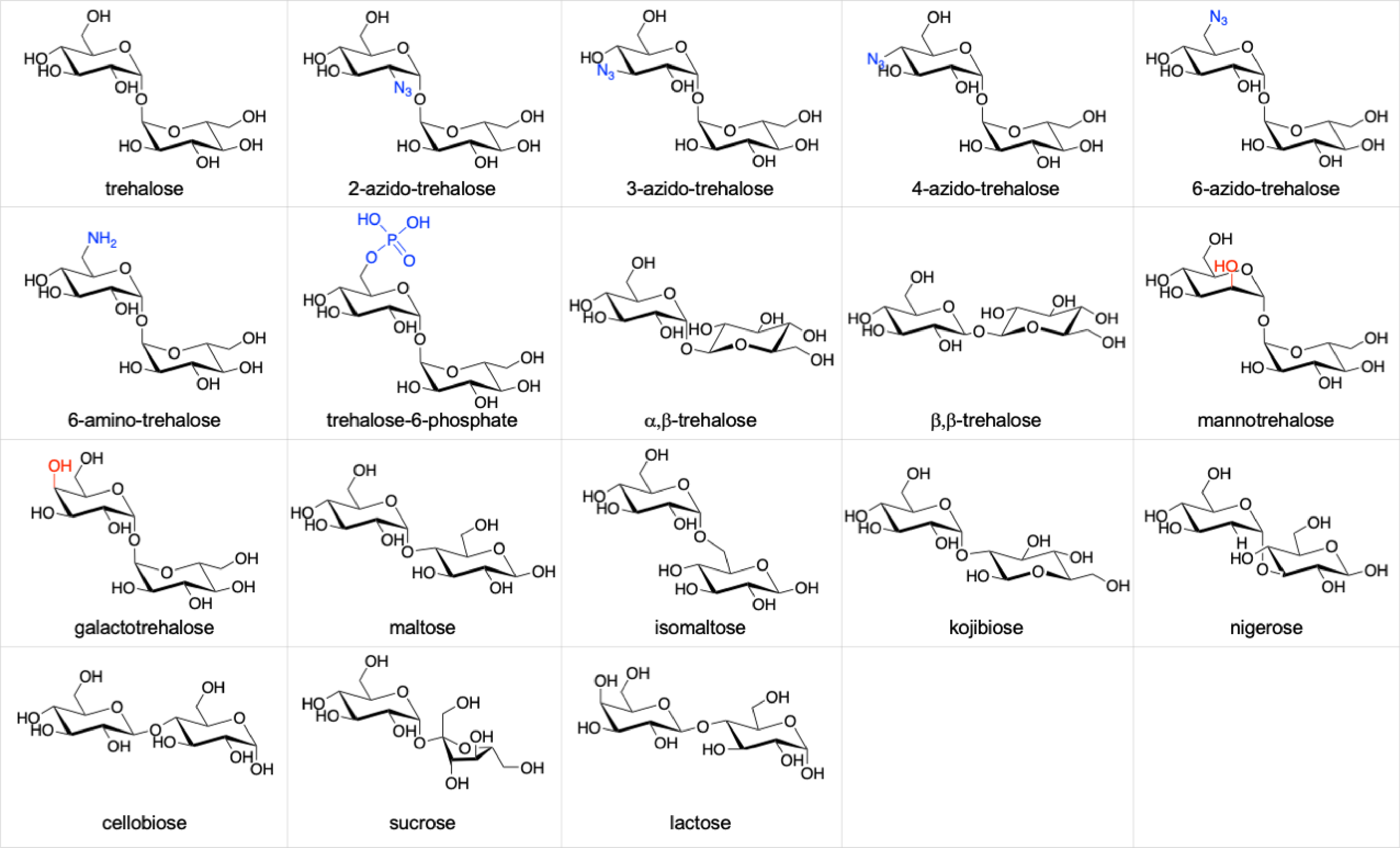

Fig. S5. ITC of *Mtr* LpqY. A) Microcalorimetric traces of trehalose injections into *Mtr* LpqY after subtraction of the control experiment (trehalose titrated into buffer) **B)** Fit of the binding isotherm for one set of binding sites. **C)** The thermodynamic parameters derived from the software using the one binding site model are shown for trehalose and galactotrehalose.

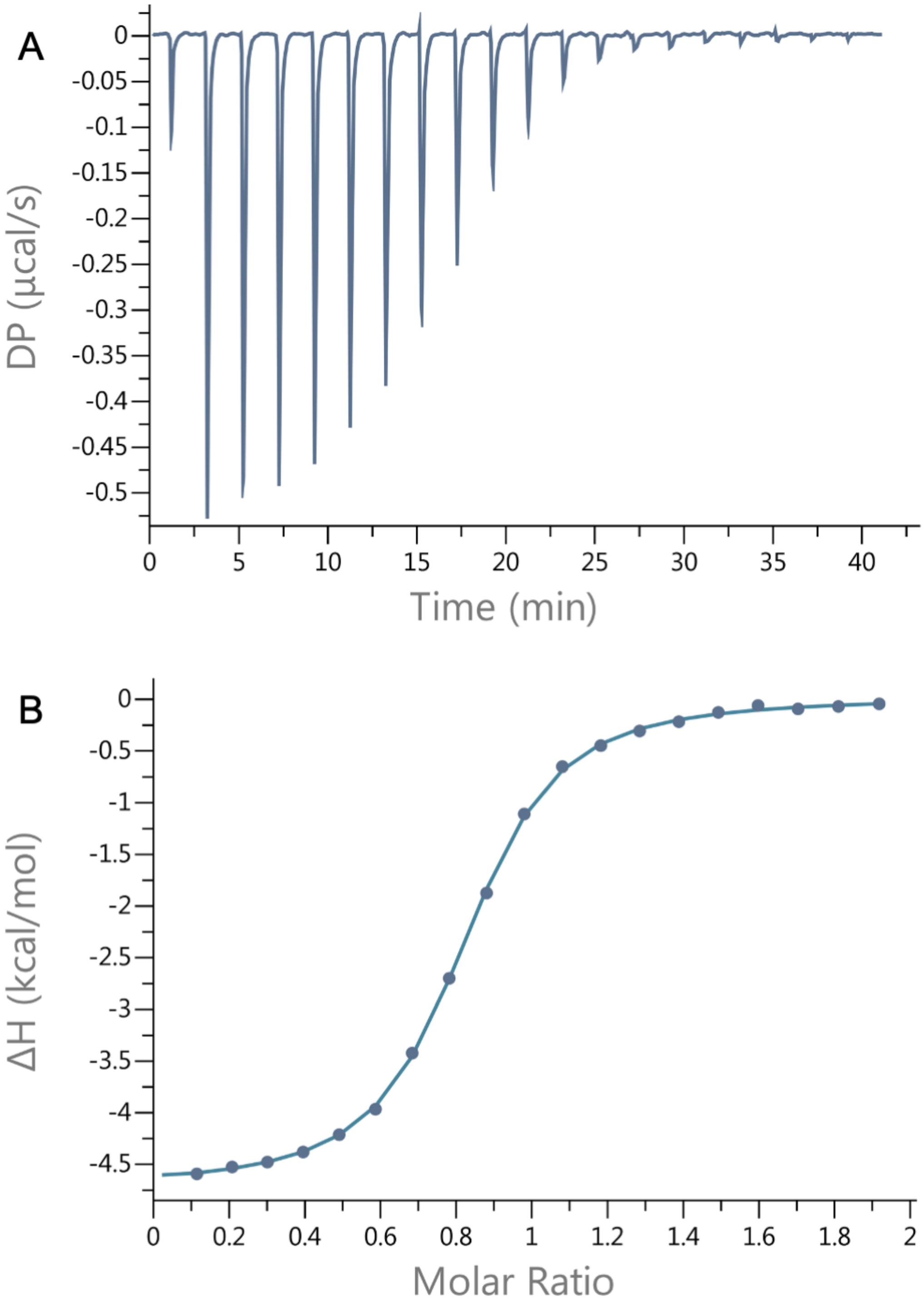

C

	Trehalose	Galactotrehalose
n	0.79 ± 0.002	1.12 ± 0.01
K_d (µM)	1.1 ± 0.04 µM	2.1 ± 0.2 µM
ΔH (kcal/mol)	- 4.7 ± 0.2	- 4.9 ± 0.1
-TΔS (kcal/mol)	- 3.42	- 2.88
ΔG (kcal/mol)	- 8.16	- 7.74

Fig. S6. Microscale thermophoresis of *Mtr* LpqY and trehalose. A) MST trace of *Mtr* LpqY with trehalose **B)** MST analysis. The bars shown in blue and red indicate the data selected for analysis.

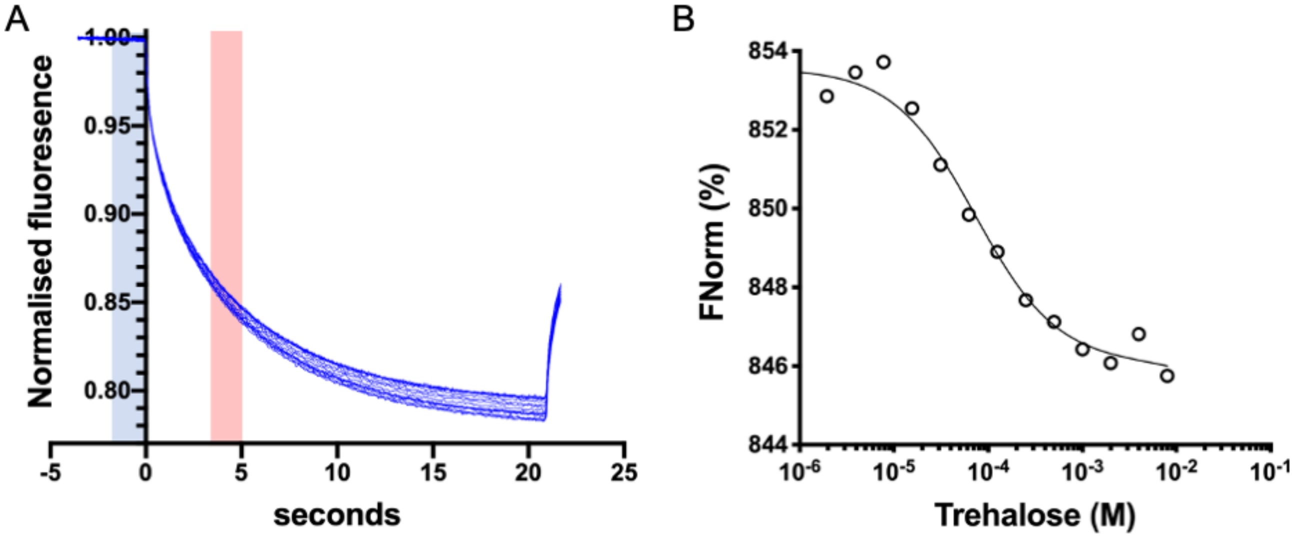

Fig. S7. Electron density for the trehalose substrate. Electron density map contoured at 0.7 electrons/Å^3. Carbon atoms are shown in green, and oxygen atoms are shown in red. The figure was prepared using CCP4mg. The .mtz file was loaded directly with the default settings and clipped to select for the trehalose atoms.

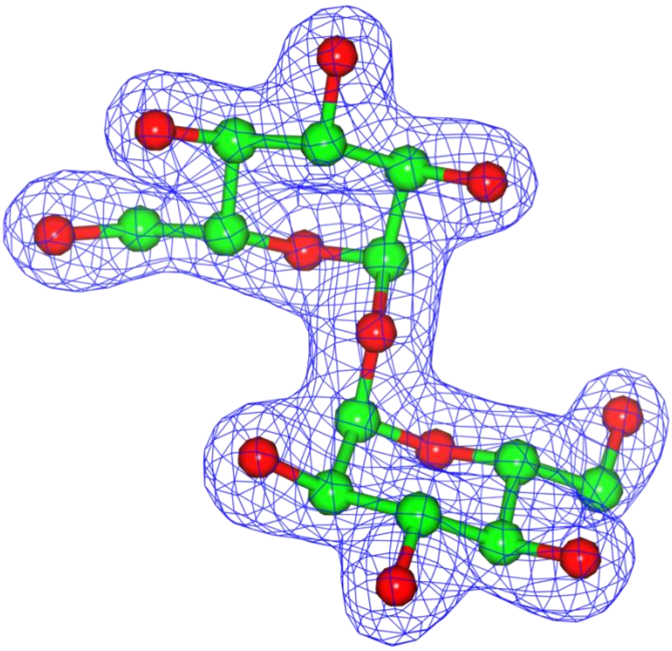

Fig. S8. Sequence alignment of mycobacterial LpqY homologues. The sequence alignment was generated using Clustal Omega (1) and ESPript version 3.0 (2). Identical residues are indicated by a red background, conserved residues by red characters and similar residues outlined in a blue box. Blue stars indicate *Mtr* LpqY residues that interact with trehalose.

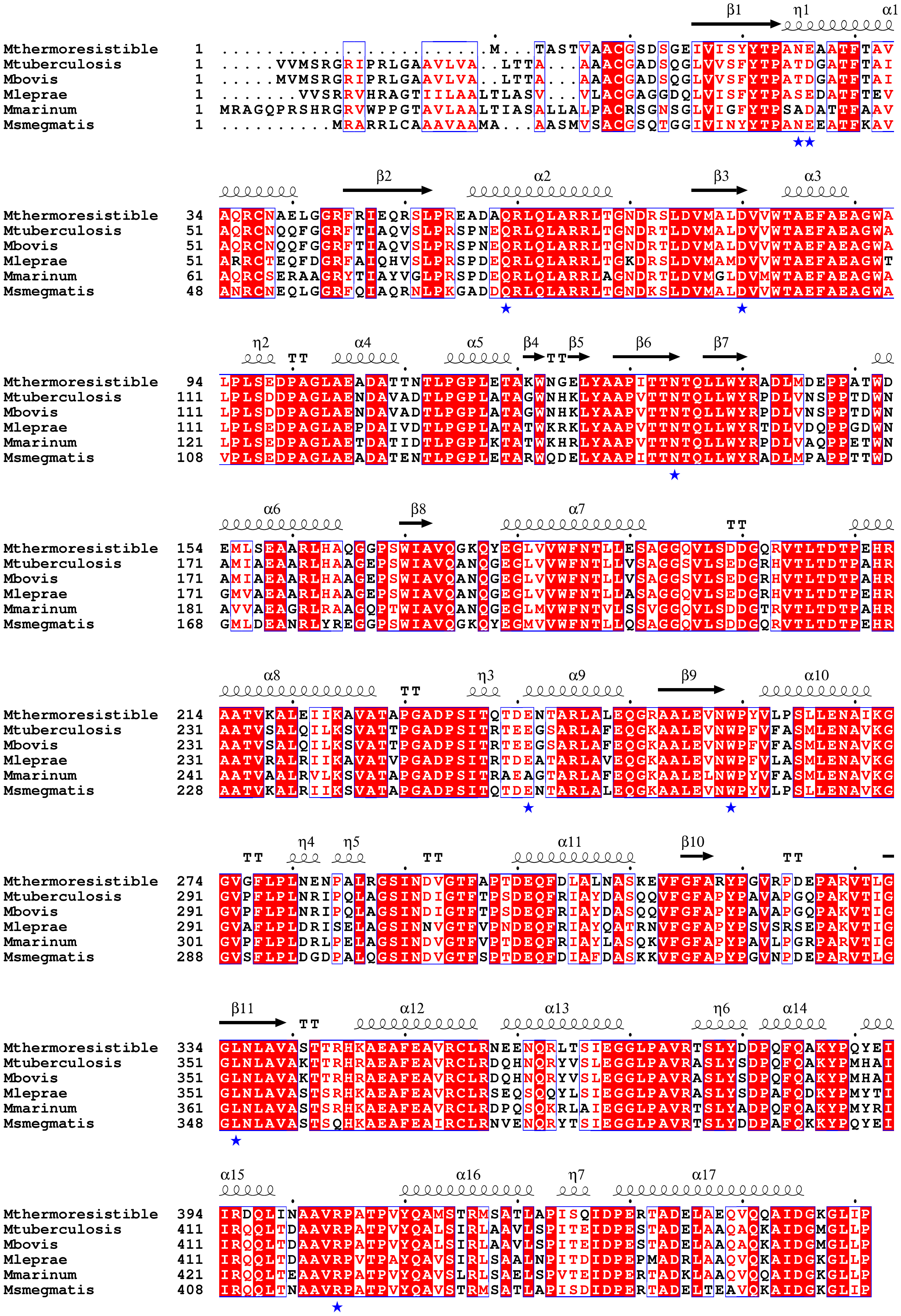

Fig S9. Circular dichroism spectra of *Mtr* LpqY and site-directed mutant proteins. CD spectra of *Mtr* LpqY (red), *Mtr* LpqY Asn25Ala (orange), *Mtr* LpqY Asn25Thr-Glu26Asp (dark green), *Mtr* LpqY Glu26Ala (yellow), *Mtr* LpqY Gln59Ala (brown), *Mtr* LpqY Asp80Ala (magenta), *Mtr* LpqY Asn134Ala (purple), *Mtr* LpqY Glu241Ala (cyan), *Mtr* LpqY Trp259Ala (green), *Mtr* LpqY Leu335Ala (blue), *Mtr* LpqY Arg404Ala (black)

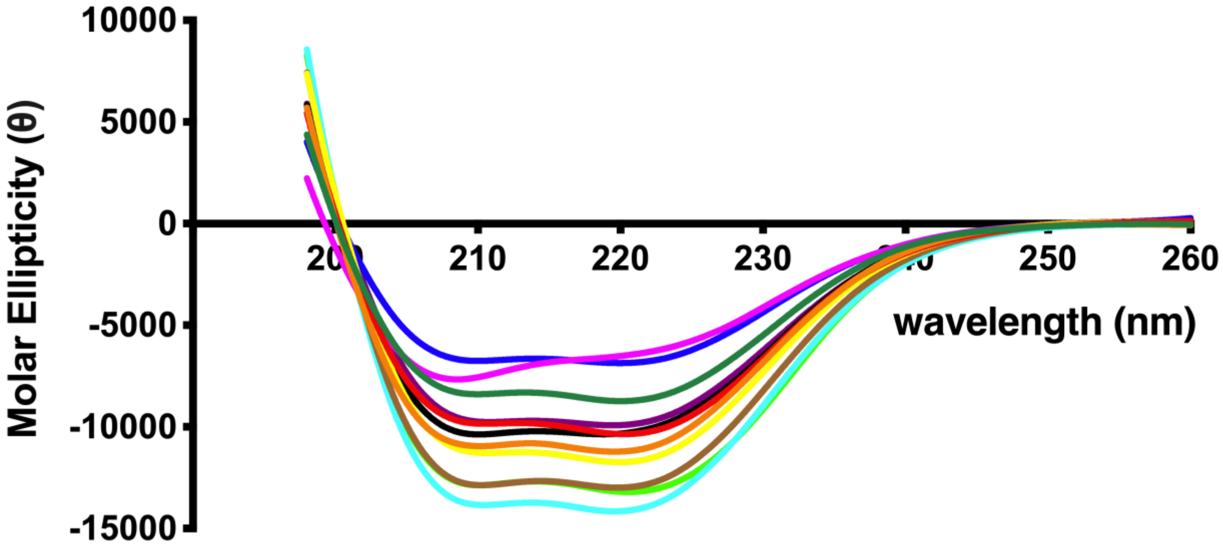

.

Fig. S10. Thermal shift assay probing a panel of potential ligands against the *Mtr* LpqY Asn25Thr-Glu26Asp double mutant. Bar graphs illustrating the ΔT_m shifts for the series of potential ligands probed for binding at a final concentration of 10 mM. Data are shown from three independent repeats represented as mean ± SD. *Mtr* LpqY is shown in black bars and *Mtr* LpqY Asn25Thr-Glu26Asp in red bars.

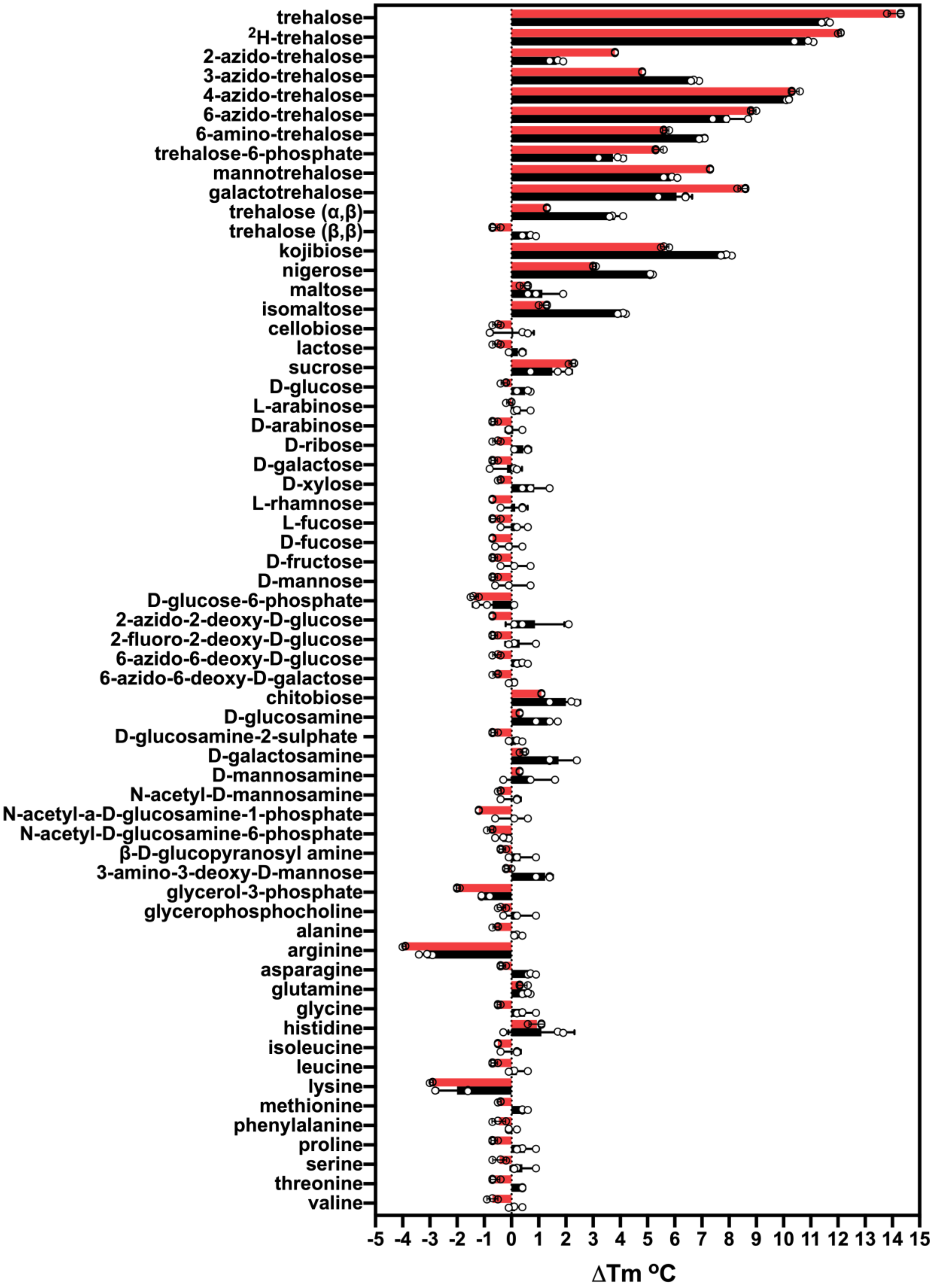

Fig. S11. Post 600 ns simulation snapshot of *Mtr* LpqY. Snapshot of *Mtr* LpqY at 0 ns, i.e., the *Mtr* LpqY-trehalose crystal structure and at 600 ns. The two domains and the hinge regions are highlighted: domain I (grey) and domain II (blue); loop 1 (cyan), loop 2 (magenta), loop 3 (orange). The trehalose ligand is represented as sticks with green carbon atoms.

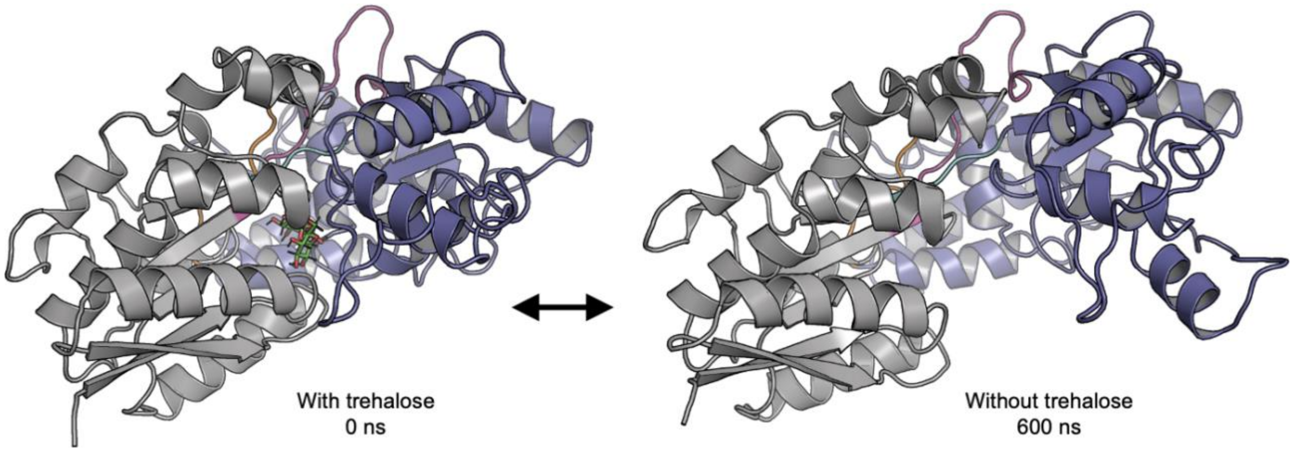

Fig. S12. Interactions of *Mtr* LpqY Asn258. A) Zoom in of the *Mtr* LpqY trehalose binding site showing trehalose and the interactions of Glu241, Asn258 and Glu256. The trehalose ligand is represented as sticks with green carbon atoms. Hydrogen bonds are shown as black dashes. **B)** Interactions of Asn258 when Glu256 protonated. **C)** Interactions of Asn258 when Glu256 is not protonated. **B)** and **C)** Molecular dynamic simulations showing the residues of *Mtr* LpqY interacting with Asn258 of the course of the simulation, where 1 is in contact for the entire simulation. Data from three repeats of 600 ns are shown, where the error bars represent standard deviation. The interactions with Glu241 and Glu256 are highlighted with red stripes.

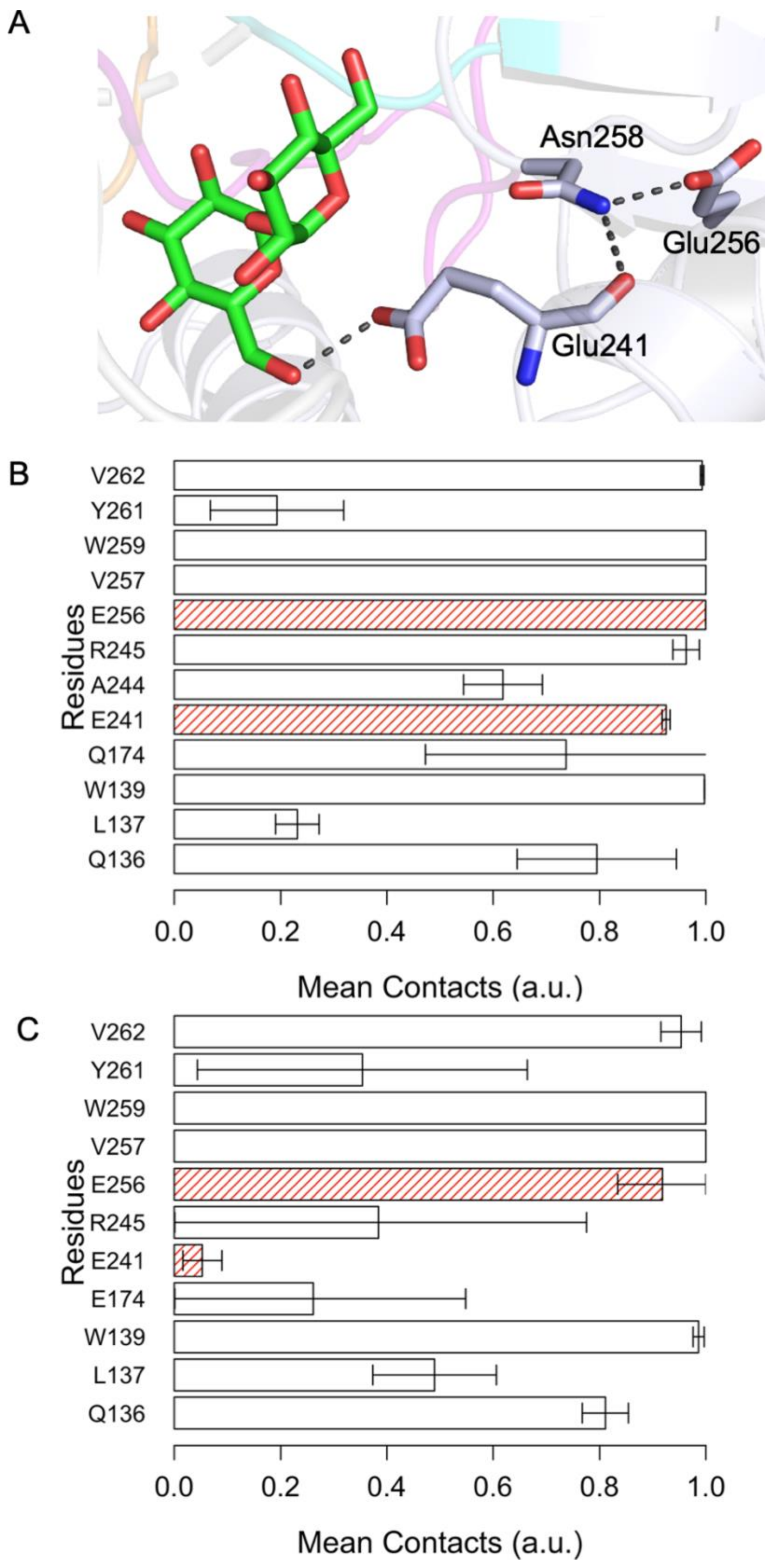

Fig. S13. STD NMR for *Mtr* LpqY with trehalose and 6-azido-trehalose. STD NMR build-up curves for trehalose (**A**) and 6-azido-6-deoxy-α,α′-trehalose (**B**) in complex with *Mtr* LpqY. Saturation frequency set at 0.80 ppm.

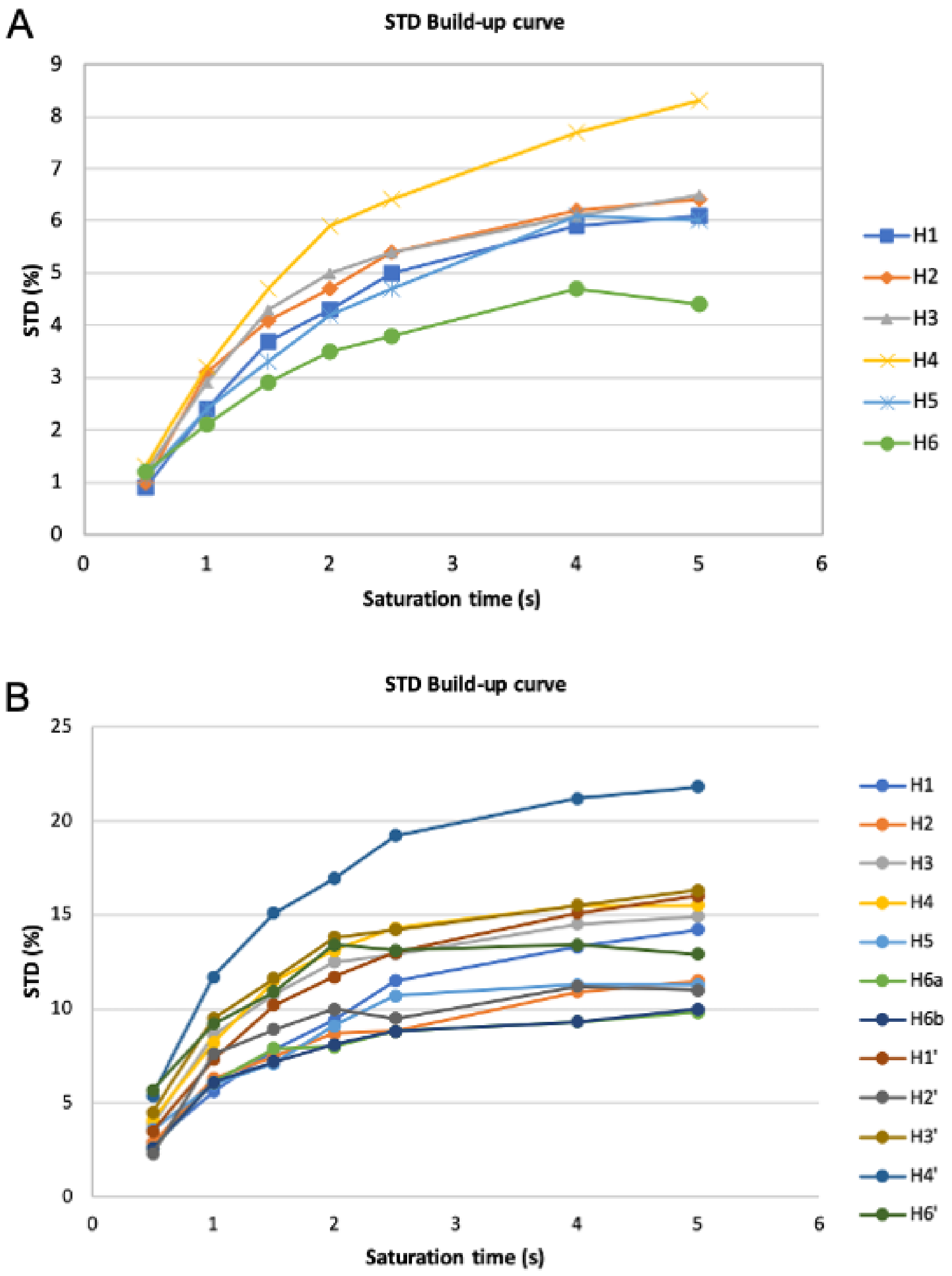

Fig. S14. Differential Epitope Mapping by STD NMR of *Mtr* LpqY with trehalose and 6-azido-trehalose. Differential Epitope Mapping histograms (0.80/7.20 ppm) for **A**) trehalose and **B**) 6-azido-6-deoxy-α,α′-trehalose in complex with *Mtr* LpqY. Positive DEEP-STD factors (ΔSTDs) after aliphatic irradiation (0.80 ppm) are shown in orange, and negative ΔSTDs after aromatic irradiation (7.20 ppm) in blue. ΔSTD values were calculated as previously described (3).

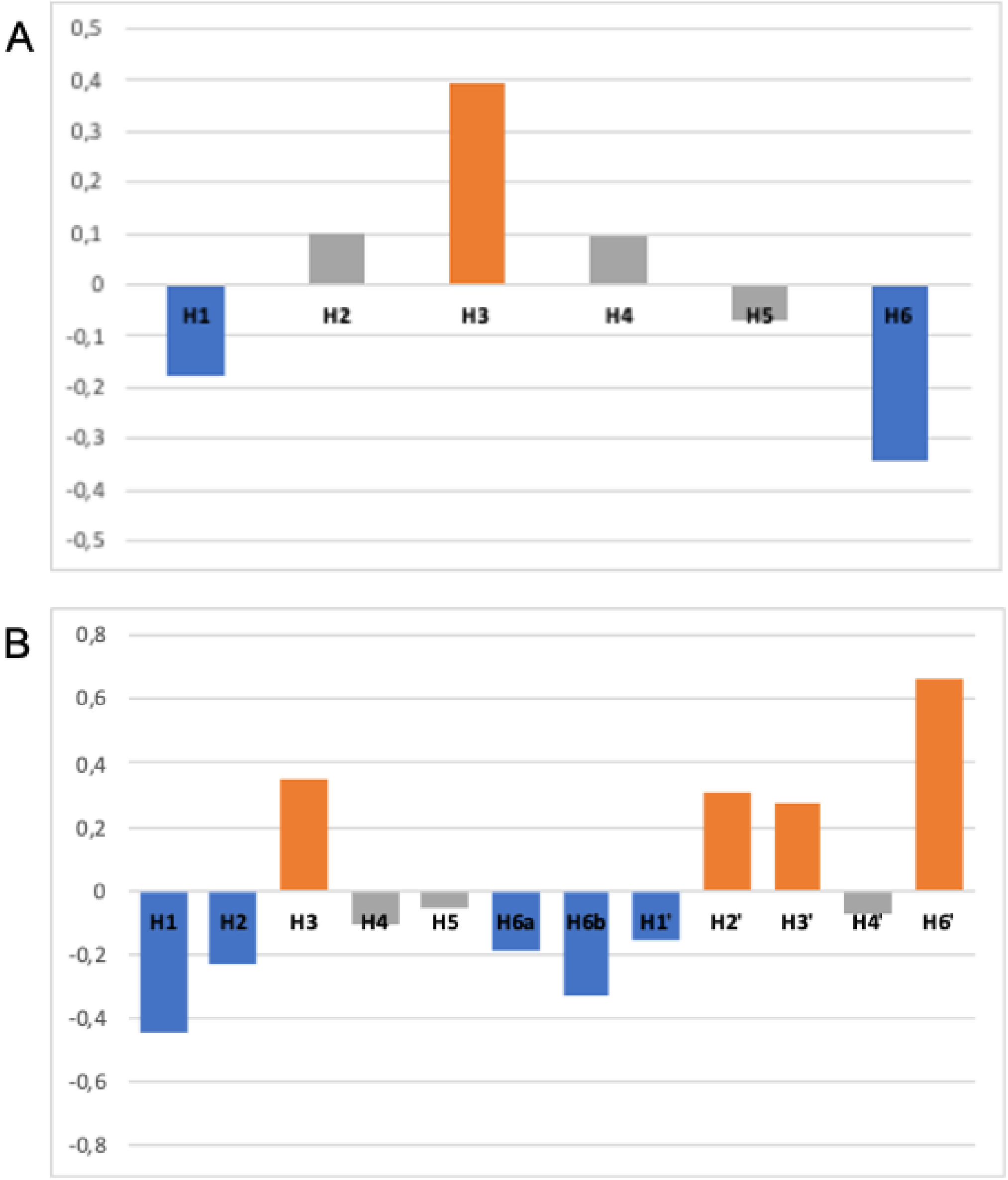

Fig. S15. Comparison of *Mtr* LpqY with *Thermus sp.* homologues A) *Mtr* LpqY (green, trehalose shown in stick representation: grey carbon atoms), **B**) *Thermus litoralis* (PDB 1EU8) (cyan, trehalose shown in stick representation: yellow carbon atoms), **C***) Thermus thermophilus* (6J9W) (blue, trehalose shown in stick representation: magenta carbon atoms), **D**) Close up superposition showing the binding orientation of the trehalose ligand. *Mtr (grey), T. litoralis* (yellow), *T. thermophilus* (magenta).

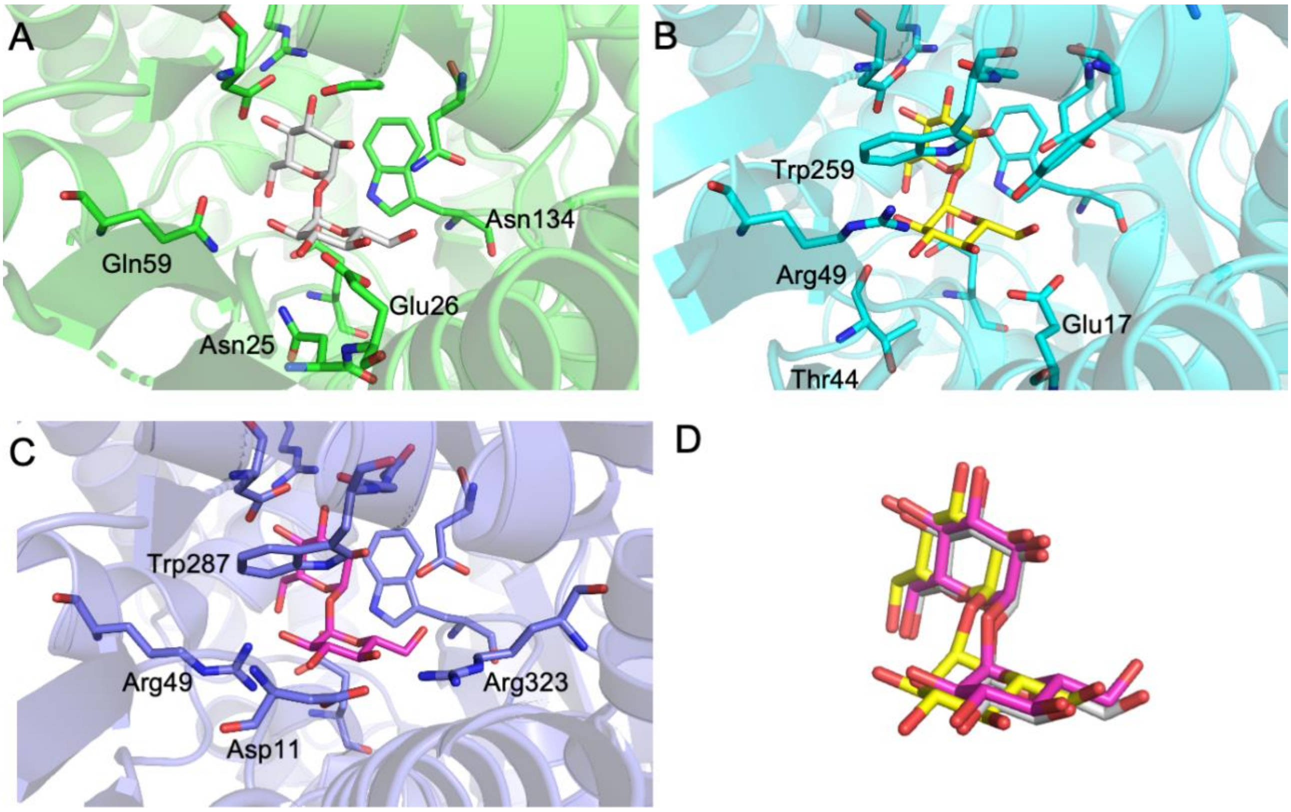

Fig. S16. Sequence alignment of *Mtr* LpqY with *Thermus sp.* homologues. The sequence alignment was generated using Clustal Omega(1) and ESPript version 3.0 (2). Identical residues are indicated by a red background, conserved residues by red characters and similar residues outlined in a blue box. Blue stars indicate *Mtr* LpqY residues that interact with trehalose

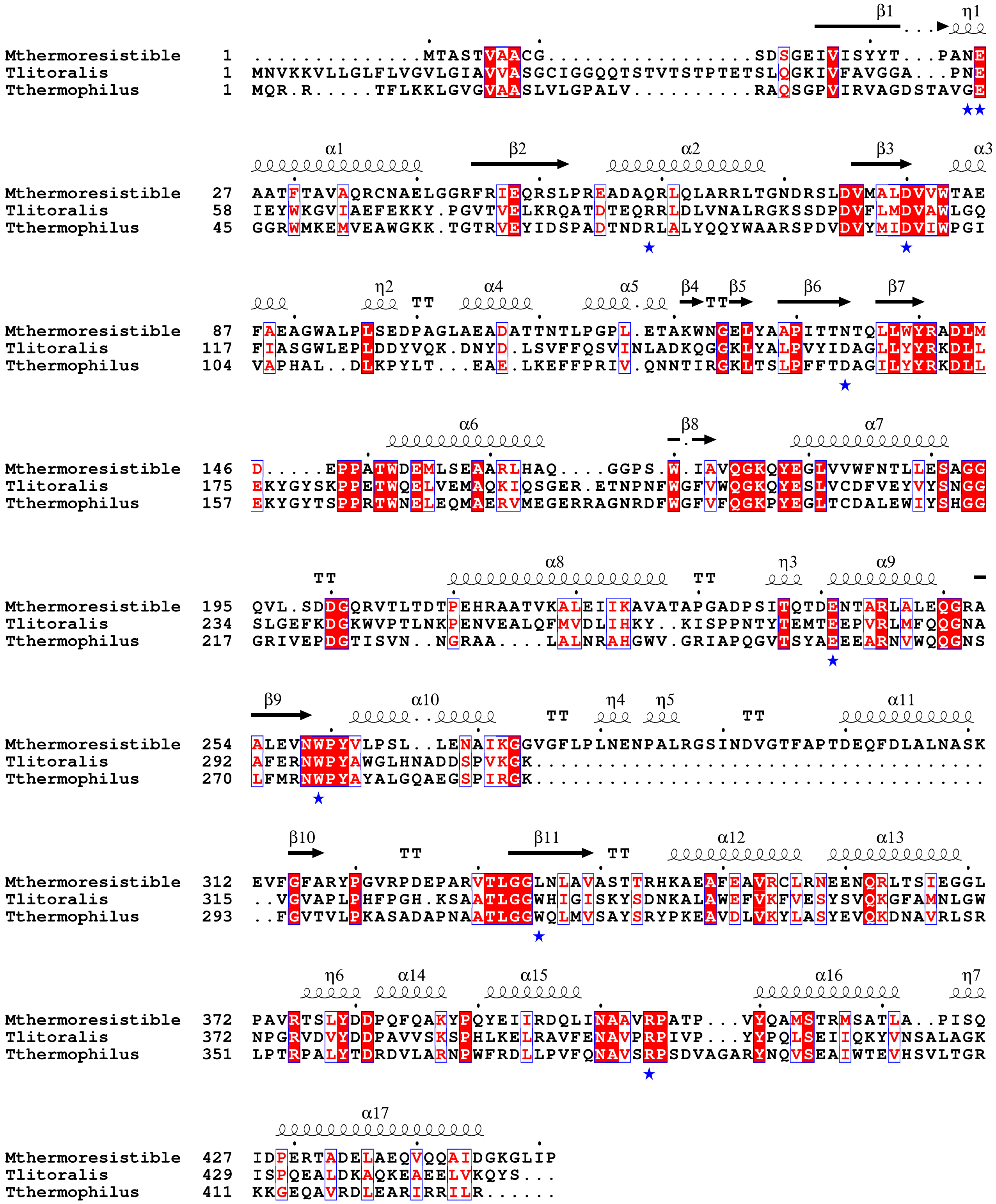

Table S1. Data collection and statistics for *Mtr* LpqY in complex with trehalose (PDB 7APE)

**PBD 7APE**	*Mtr* LpqY-trehalose
**Data Collection**	
Beam line	I03
Wavelength (Å)	0.98
Space group	$P4_12_12_1$
Unit cell parameters	
a (Å)	92.4
b (Å)	92.4
c (Å)	216.6
α	90
β	90
γ	90
Molecules in ASU	2
Resolution (Å)	48.4 - 1.7
(Outer shell)[a]	1.76 - 1.7
Unique reflections	102,435 (9,278)
Multiplicity	23.0 (10.7)
$CC_{1/2}$	0.999 (0.479)
Completeness (%)[a]	98.7 (90.9)
R_{merge} [a]	0.11 (1.85)
Mean I/σ(I)[a]	15.2 (1.2)
**Refinement**	
R work (%)	16.9
R free (%)	19.4
r.m.s.d.	
Bond lengths (Å)	0.008
Bond angles (degrees)	0.87
No. of non-hydrogen atoms	7,414
Protein atoms	6,662
Ligand	46
Solvent waters	706
Average B factors ($Å^2$)	
Overall	30.6
Protein	29.7
Ligand	21.5
Solvent	39.9
Ramachandran plot[b]	
Favoured region (%)	97.7
Allowed region (%)	2.3
Outer region (%)	0.00

[a] Highest-resolution shell in parenthesis
[b] Ramachandran plot statistics were calculated by MolProbity

Table S2. Sequence of primers for cloning and site-directed mutagenesis
Restriction recognition sites are in italics. The codon encoding the amino acid mutation is indicated in bold type.

Name	Use	Sequence (5'-3')
LpqY_SUMO_5	Clone LpqY into pETSUMO	aaaa*ggatcc*atgacggcctcgacggtcg
LpqY_SUMO_3	Clone LpqY into pETSUMO	aaaa*aagctt*tcatgggatcagccccttaccg
Asn25Ala_F	Mutate LpqY residue Glu26Ala	cagctactacacgccggca**gcc**gaggcggccacgttcactg
Asn25Ala_R	Mutate LpqY residue Glu26Ala	cagtgaacgtggccgcctc**ggc**tgccggcgtgtagtagctg
Asn25Thr_Glu26Asp_F	Mutate LpqY residue Glu26Thr and Glu26Asp	agctactacacgccggca**accgat**gcggccacgttcactgctg
Asn25Thr_Glu26Asp_R	Mutate LpqY residue Glu26Thr and Glu26Asp	cagcagtgaacgtggccgc**atcggt**tgccggcgtgtagtagctg
Glu26Ala_F	Mutate LpqY residue Glu26Ala	ctacacgccggcaaac**gcg**gcggccacgttcactg
Glu26Ala_R	Mutate LpqY residue Glu26Ala	cagtgaacgtggccgc**cgc**gtttgccggcgtgtag
Gln59Ala_F	Mutate LpqY residue Gln59Ala	cgcgcgaggccgacgcc**gcg**cggttgcagctggctc
Gln59Ala_R	Mutate LpqY residue Gln59Ala	gagccagctgcaaccg**cgc**ggcgtcggcctcgcgcg
Asp80Ala_F	Mutate LpqY residue Asp80Ala	ggacgtgatggcgctc**gcg**gtggtgtggaccgccg
Asp80Ala_R	Mutate LpqY residue Asp80Ala	cggcggtccacaccac**cgc**gagcgccatcacgtcc
Asp134Ala_F	Mutate LpqY residue Asp134Ala	gcgcccatcaccacc**gcc**acccagttgctctgg
Asp134Ala_R	Mutate LpqY residue Asp134Ala	ccagagcaactgggt**ggc**ggtggtgatgggcgc
Glu241Ala_F	Mutate LpqY residue Glu241Ala	gatcacccagaccgac**gcg**aacaccgctcggttgg
Glu241Ala_R	Mutate LpqY residue Glu241Ala	ccaaccgagcggtgtt**cgc**gtcggtctgggtgatc
Trp259Ala_F	Mutate LpqY residue Trp259Ala	gcgctggaggtgaac**gcg**ccctacgtgttgccg
Trp259Ala_R	Mutate LpqY residue Trp259Ala	cggcaacacgtaggg**cgc**gttcacctccagcgc
Leu335Ala_F	Mutate LpqY residue Leu335Ala	gggtcaccctgggcggg**gcg**aacctggcggtggccag
Leu335Ala_R	Mutate LpqY residue Leu335Ala	ctggccaccgccaggtt**cgc**cccgcccagggtgaccc
Arg404Ala_F	Mutate LpqY residue Arg404Ala	gatcaacgccgcggtg**gcc**ccggcgaccccggtc
Arg404Ala_R	Mutate LpqY residue Arg404Ala	gaccggggtcgccgg**ggc**caccgcggcgttgatc

Synthetic Methods

NMR spectroscopy (^{1}H, ^{13}C) was conducted on either a Bruker DPX-400 or Bruker DPX-500 spectrometer at 298 K, and all chemical shifts (δ) are given in ppm relative to the solvent reference. Coupling constants (J) are reported in hertz (Hz) with the following abbreviations: s, singlet; d, doublet; t, triplet; q, quartet; quin, quintet; m, multiplet; br, broad. TLCs were performed on Merck silica gel 60 F-254 TLC sheets. Flash chromatography was carried out using Sigma–Aldrich technical grade silica gel (pore size, 60 Å; particle size, 40–63 μm) as the stationary phase. Mass spectra were recorded on a Bruker Esquire 2000 spectrometer using electrospray ionisation (ESI). *m/z* values are reported in Daltons (Da).

^{2}H-trehalose

Trehalose dihydrate (1.13 g, 3.0 mmol) was dissolved into 4 mL of D_2O to which Ru/C (5 wt %, 606 mg, 0.3 mmol) was added. The solution was stirred in a crimp sealed reaction vessel, degassed with hydrogen, after which a balloon filled with hydrogen gas was fitted to the vessel, to maintain a pressure of ~ 1 bar. The vessel was subsequently heated to 80 °C for 72 h. After this time the reaction was cooled to room temperature, filtered through a 0.2 μm membrane filter and concentrated *in vacuo*. An NMR of this crude material showed approximately 70 % deuteration. The crude material was re-dissolved in D_2O (16 mL) and a further portion of Ru/C (5 wt %, 404 mg, 0.2 mmol) was added. The vessel was sealed with a new crimp seal, degassed with hydrogen, after which a balloon filled with hydrogen gas was fitted to the vessel, to maintain a pressure of ~ 1 bar. The vessel was heated to 80 °C for a further 48 h, then cooled, filtered (0.2 μm membrane filter), and the filtrate lyophilised to give the product as a white solid (657 mg, 54 %). NMR showed 93 % deuteration at the 2,3,4 and 6 positions. ^{1}H NMR (400 MHz, D_2O) δ_{ppm} 5.09 (2H, s, H^1), 3.71 (2H, s, H^5). ^{13}C NMR (100 MHz, D_2O) δ_{ppm} 93.1 (C^1), 71.9 (C^5), 70.6 (m), 69.2 (m), 59.8 (m) (C^2, C^3, C^4, C^6). *m/z* (ES^-): $[M-H]^-$ calcd. for $C_{12}H_{11}D_{10}O_{11}^-$, 351.2; found 351.1.

6-bromo-6-deoxy-α,α′-trehalose

Anhydrous trehalose (2 g, 5.84 mmol), triphenylphosphine (3.06 g, 11.7 mmol) and *N*-bromosuccinimde (2.08 g, 11.7 mmol) were dissolved in anhydrous DMF (20 mL) under nitrogen and the reaction mixture was heated to 60 °C for 18 h. The reaction mixture was then cooled to room temperature and concentrated *in vacuo* to give a brown oil which was purified by column chromatography (100:0 – 70:30 EtOAc/MeOH) to give the mono-brominated product as an off white solid (780 mg, 33 %). ^{1}H NMR (500MHz, D_2O) δ_{ppm} 5.12 (1H, d, *J* = 3.5 Hz, H^1), 5.11 (1H, d, *J* = 4.0 Hz, $H^{1'}$), 3.90 (1H, ddd, *J* = 9.5 Hz, 5.5 Hz, 2.0 Hz, H^5), 3.64 – 3.81 (6H, m, $H^{6ab'}$, $H^{5'}$, H^3, $H^{3'}$, H^{6a}), 3.53 – 3.62 (3H, m, H^{6b}, H^2, $H^{2'}$), 3.41 (1H, app t, *J* = 9.5 Hz, H^4), 3.36 (1H, app t, *J* = 9.5 Hz, $H^{4'}$).^{13}C NMR (100 MHz, D_2O) δ_{ppm} 93.5, 93.3 (C^1 + $C^{1'}$), 72.5, 72.2, 72.2, 71.6, 70.9, 70.7, 69.6 (C^2, C^3, C^4, C^5, $C^{2'}$, $C^{3'}$, $C^{4'}$, $C^{5'}$), 60.5 ($C^{6'}H_2OH$), 33.0 (C^6H_2Br). (ES^-): $[M + H_2O – H]^-$ calcd. for $C_{12}H_{23}BrO_{11}^-$ 422.0, 424.0; found 421.1, 423.1.

6-azido-6-deoxy-α,α′-trehalose

6-bromo-6-deoxy-α,α′-trehalose (780 mg, 1.93 mmol) was dissolved in anhydrous DMF (20 mL) and sodium azide (751 mg, 11.6 mmol) was added portion-wise. The reaction mixture was then heated to 95 °C for 72 h before being cooled to room temperature and filtered. The filtrate was concentrated *in vacuo* to give an orange solid (1.2 g), which was purified by column chromatography (5:3:1 EtOAc/*n*-PrOH/H_2O) to give the product as a yellow solid (491 mg, 69 %). ^{1}H NMR (500MHz, D_2O) δ_{ppm} 5.10 (2H, app t, *J* = 4.5 Hz, H^1 + $H^{1'}$), 3.87 (1H, ddd, *J* = 9.0, 6.0, 2.5 Hz), 3.70 – 3.80 (4H, m), 3.67 (1H, dd, *J* = 12.0, 5.0 Hz), 3.53 – 3.62 (3H, m), 3.47 (1H, dd, *J* = 13.5, 6.0 Hz), 3.36 (2H, td, *J* = 9.5, 3.5 Hz). ^{13}C NMR (126 MHz, D_2O) δ_{ppm} 93.6, 93.4 (C^1 and $C^{1'}$), 72.5, 72.3, 72.2, 71.0, 70.9, 70.9, 70.4, 69.6 (C^2, C^3, C^4, C^5, $C^{2'}$, $C^{3'}$, $C^{4'}$, $C^{5'}$), 60.5 ($C^{6'}H_2OH$), 50.8 ($C^6H_2N_3$). *m/z* (ES^-): $[M-H]^-$ calcd. for $C_{12}H_{20}N_3O_{10}^-$, 366.3; found 366.2.

6-amino-6-deoxy-α,α′-trehalose

H_2NNH_2 (51 wt %, 85.5 μL, 1.36 mmol) was added to 6-azido-6-deoxy-α,α′-trehalose (200 mg, 0.544 mmol) and $Pd(OH)_2$/C (20 wt %, 38.2 mg, 0.0544 mmol) in methanol (20 mL) under nitrogen. The reaction mixture was refluxed for 16 h, before being cooled to room temperature, filtered and concentrated *in vacuo* to give the product as a pale brown solid (173 mg, 93 %). ^{1}H NMR (400 MHz, D_2O) δ_{ppm} 5.12 (2H, d, *J* = 3.5 Hz, H^1, $H^{1'}$), 3.64 – 3.81 (6H, m, H^3, $H^{3'}$, $H^{6ab'}$, H^5, $H^{5'}$), 3.57 (2H, dd, *J* = Hz, H^2, $H^{2'}$), 3.37 (1H, app t, *J* = 9.5 Hz, $H^{4'}$), 3.26 (1H, app t, *J* = 9.5 Hz, H^4), 3.01 (1H, dd, *J* = 13.5 Hz, 2.5 Hz, H^{6a}), 2.75 (1H, dd, *J* = 13.5 Hz, 8.0 Hz, H^{6b}). ^{13}C NMR (100 MHz, D_2O) δ_{ppm} 93.2, 93.1 (C^1, $C^{1'}$), 72.5, 72.4, 72.2, 71.6, 71.6, 71.4, 71.0, 69.6 (C^2, C^3, C^4, C^5, $C^{2'}$, $C^{3'}$, $C^{4'}$, $C^{5'}$), 60.5 ($C^{6'}H_2OH$), 41.3 ($C^6H_2NH_2$). *m/z* (ES^-) $[M-H]^-$ calcd. for $C_{12}H_{22}NO_{10}^-$, 340.1; found 340.1.

2,3,6,2′,3′,4′,6’,-hepta-*O*-benzoyl-α,α’-D-trehalose

Trehalose dihydrate (5 g, 13.2 mmol) was suspended in pyridine (75 mL) under nitrogen and cooled to -40 °C. Benzoyl chloride (11.5 mL, 99.1 mmol) was added dropwise and the reaction was maintained at -40 °C for 2 h before allowing to warm slowly to room temperature and stirred for 16 h. A further portion of benzoyl chloride was added (1.54 mL, 13.2 mmol) and the reaction stirred at room temperature for a further 20 h. The reaction mixture was poured into ice cold 1 M HCl (100 mL) and extracted with EtOAc (3 x 80 mL). The combined organics extracts were washed with sat. $NaHCO_3$ (2 x 80 mL) and brine (80 mL). The organic phase was dried ($MgSO_4$), filtered and concentrated *in vacuo* to give the crude product which was purified by column chromatography (9:1, toluene/EtOAc) to give the desired product as a white foam (1.95g, 14 %). ^{1}H NMR (400 MHz, CDCl3) δ 7.81 – 8.13 (14H, m, ArH), 7.22 – 7.62 (21H, m, ArH), 6.28 (1H, t, *J* = 10.0 Hz, H^3), 5.98 (1H, t, *J* = 9.5 Hz, $H^{3'}$), 5.68 – 5.71 (2H, m, H^1, H^4), 5.64 (1H, d, *J* = 4.0 Hz, H1’), 5.50 (1H, dd, *J* = 10.5, 4.0 Hz, H^2), 5.46 (1H, dd, *J* = 10.0, 4.0 Hz, $H^{2'}$), 4.37 (1H, ddd, *J* = 10.5, 4.5, 3.0 Hz, H^5), 4.22 (1H, dd, *J* = 12.5, 4.0 Hz, $H^{6a'}$), 4.08 (1H, ddd, *J* = 10.0, 4.0, 2.0 Hz, $H^{5'}$), 3.98 (1H, dd, *J* = 12.5, 3.0 Hz, H^{6a}), 3.85 – 3.91 (2H, m, H^{6b}, $H^{6b'}$), 3.82 (1H, t, *J* = 10.0 Hz, $H^{4'}$); ^{13}C NMR (100 MHz, CDCl3) δ 167.3, 167.0, 165.9, 165.7, 165.5, 165.0 (C=O), 134.1, 133.9, 133.6, 133.60, 133.5, 133.3, 133.2, 130.3, 130.0, 130.0, 130.0, 129.9, 129.9, 129.8, 129.5, 129.4, 129.2, 129.1, 128.9, 128.8, 128.8, 128.6, 128.5, 128.5, 128.4 (ArC), 93.1, 92.9(C^1, $C^{1'}$),

73.6, 71.3, 71.2, 70.8, 70.3, 69.3, 68.9, 68.7 (C^2, C^3, C^4, C^5, $C^{2'}$, $C^{3'}$, $C^{4'}$, $C^{5'}$), 62.5, 62.0 (C^6, $C^{6'}$); *m/z* (ES^+): $[M+Na]^+$ calcd. for $C_{61}H_{50}O_{18}Na^+$, 1093.3; found 1093.3.

2,3,6,-tri-*O*-benzoyl-α-D-galactopyranosyl-(1→1)-2′,3′,4′,6′,-tetra-*O*-benzoyl-α-D-glucopyranoside

2,3,6,2′,3′,4′,6,-Hepta-*O*-benzoyl-α,α-D-trehalose (1.95 g, 1.82 mmol) was dissolved in DCM (30 mL) under nitrogen and cooled to 0 °C. Pyridine (1.47 mL, 18.2 mmol) and triflic anhydride (613 μL, 3.64 mmol) were added and the reaction allowed to slowly warm to room temperature and stirred for 3 h. The reaction mixture was diluted with DCM (20 mL) and washed with 1 M HCl (50 mL), sat. $NaHCO_3$ (50 mL) and water (50 mL). The organic phase was dried ($MgSO_4$), filtered and concentrated *in vacuo* to give the intermediate triflate as a white solid, which was dissolved in DMF (16 mL) under nitrogen and sodium nitrite (629 mg, 9.11 mmol) was added. The reaction was stirred at room temperature for 16 h. A further portion of sodium nitrite (251 mg, 3.64 mmol) was added and the reaction stirred for a further 6 h. The reaction was diluted with DCM (60 mL) and washed with water (4 x 60 mL). The organic phase was dried ($MgSO_4$), filtered and concentrated *in vacuo* to give the crude product which was purified by column chromatography (9:1, toluene/EtOAc) to give the desired product as a white solid (670 mg, 34 %). ^{1}H NMR (400 MHz, CDCl3) δ 7.73 – 8.14 (14H, m, Ar<u>H</u>), 7.20 – 7.63 (21H, m, Ar<u>H</u>), 6.25 (1H, t, *J* = 10.0 Hz, <u>H</u>^{3}), 5.85 – 5.97 (2H, m, $H^{2'}$, $H^{3'}$), 5.75 (1H, d, *J* = 4.0 Hz, H^1), 5.72 (1H, d, *J* = 3.0 Hz, $H^{1'}$), 5.65 (1H, t, *J* = 10.0 Hz, H^4), 5.47 (1H, dd, *J* = 10.0, 4.0 Hz, H^2), 4.18 – 4.38 (4H, m, H^4, H^5, $H^{5'}$, $H^{6a'}$), 3.99 – 4.08 (2H, m, H^{6a}, $H^{6b'}$), 3.94 (1H, dd, *J* = 12.5, 5.0 Hz, H^{6b}); ^{13}C NMR (100 MHz, CDCl3) δ 166.2, 166.0, 165.8, 165.7, 165.7, 165.5, 165.1 (<u>C</u>=O), 133.8, 133.7, 133.7, 133.6, 133.4, 133.3, 133.2, 130.0, 130.0, 129.9, 129.9, 129.8, 129.5, 129.4, 129.3, 129.2, 129.0, 128.8, 128.8, 128.8, 128.7, 128.6, 128.5, 128.5, 128.4 (Ar<u>C</u>), 93.1, 92.4 (C^1, $C^{1'}$), 71.4, 70.8, 70.4, 69.0, 68.6, 68.6, 68.2, 67.5 (C^2, C^3, C^4, C^5, $C^{2'}$, $C^{3'}$, $C^{4'}$, $C^{5'}$), 62.35, 62.18 (C^6, $C^{6'}$); *m/z* (ES^+): $[M+Na]^+$ calcd. for $C_{61}H_{50}O_{18}Na^+$, 1093.3; found 1093.3.

4-Azido-2,3,6,-tri-*O*-benzoyl-α-D-galactopyranosyl-(1→1)-2′,3′,4′,6′,-tetra-*O*-benzoyl-α-D-glucopyranoside

2,3,6,-tri-O-benzoyl-α-D-galactopyranosyl-(1→1)-2′,3′,4′,6′,-tetra-O-benzoyl-α-D-glucopyranoside (460 mg, 0.430 mmol) was dissolved in DCM (10 mL) under nitrogen and cooled to 0 °C. Pyridine (348 μL, 4.30 mmol) and triflic anhydride (145 μL, 0.860 mmol) were added and the reaction allowed to slowly warm to room temperature and stirred for 2 h. The reaction mixture was diluted with DCM (15 mL) and washed with 1 M HCl (30 mL), sat. NaHCO3 (30 mL) and water (30 mL). The organic phase was dried ($MgSO_4$), filtered and concentrated in vacuo to give the intermediate triflate as a white solid, which was dissolved in DMF (10 mL) under nitrogen and sodium azide (98 mg, 1.50 mmol) was added. The reaction was stirred at 90 °C for 16 h. The reaction concentrated in vacuo and the residue taken up in DCM (40 mL) and washed with water (3 x 50 mL). The organic phase was dried ($MgSO_4$), filtered and concentrated in vacuo to give the crude product which was purified by column chromatography (9:1, toluene/EtOAc) to give the desired product as a white solid (410 mg, 87 %). ^{1}H NMR (400 MHz, CDCl3) δ 7.78 – 8.12 (14H, m, ArH), 7.17 – 7.66 (21H, m, ArH), 6.25 (1H, t, *J* = 10.0 Hz, $H^{3'}$), 6.14 (1H, t, *J* = 10.0 Hz, H^3), 5.60 – 5.74 (3H, m, H^1, $H^{1'}$, $H^{4'}$), 5.47 (1H, dd, *J* = 10.5, 4.0

Hz, $H^{2'}$), 5.38 (1H, dd, *J* = 10.0, 4.0 Hz, H^2), 4.25 – 4.36 (1H, m, $H^{5'}$), 3.89 – 4.08 (4H, m, H^5, H^{6a}, H^{6b}, $H^{6a'}$), 3.77 – 3.89 (2H, m, H^4, $H^{6b'}$); ^{13}C NMR (100 MHz, $CDCl_3$) δ ^{13}C NMR (100 MHz, $CDCl_3$) δ 165.8, 165.8, 165.5, 165.4, 165.4, 164.9 (C=O), 134.1, 133.9, 133.6, 133.5, 133.4, 133.2, 133.1, 129.9, 129.9, 129.8, 129.8, 129.7, 128.9, 128.7, 128.6, 128.5, 128.4, 128.4 (ArC), 93.1, 93.0 (C^1, $C^{1'}$), 71.1, 70.9, 70.1, 68.9, 68.7, 68.6 (C^2, C^3, C^5, $C^{2'}$, $C^{3'}$, $C^{4'}$, $C^{5'}$), 62.3, 61.8 (C^6, $C^{6'}$), 60.5 (C^4). *m/z* (ES^+): $[M+Na]^+$ calcd. for $C_{61}H_{49}O_{17}N_3Na^+$, 1118.3; found 1118.3.

4-Azido-4-deoxy-α,α′-trehalose

4-Azido-2,3,6,-tri-O-benzoyl-α-D-galactopyranosyl-(1→1)-2′,3′,4′,6′,-tetra-O-benzoyl-α-D-glucopyranoside (410 mg, 0.374 mmol) was dissolved in 0.2 M methanolic NaOMe (10 mL) and the reaction was stirred at room temperature for 14 h. Amberlite® IR120 acidic resin (200 mg) was then added and the mixture stirred at room temperature for 30 mins to neutralise the reaction, filtered and concentrated in vacuo. The residue was taken up in water (20 mL), washed with petroleum ether (40-60 °C) and then lyophilised. The crude product was purified by column chromatography (2.5:1 CHCl3/MeOH) to give the product as a white solid (103 mg, 75 %); 1H NMR (400 MHz, D_2O) δ 5.13 (1H, d, *J* = 4.0 Hz, H^1), 5.08 (1H, d, *J* = 4.0 Hz, $H^{1'}$), 3.91 (1H, t, *J* = 9.5 Hz, H^3), 3.65 – 3.82 (7H, m, $H^{3'}$, H^5, $H^{5'}$, H^{6a}, H^{6b}, $H^{6a'}$, $H^{6b'}$), 3.63 (1H, dd, *J* = 10.0, 4.0 Hz, H^2), 3.54 (1H, dd, *J* = 10.0, 4.0 Hz, $H^{2'}$), 3.41 (1H, t, *J* = 9.5 Hz, H^4), 3.36 (1H, t, *J* = 9.5 Hz, $H^{4'}$). ^{13}C NMR (100 MHz, D_2O) δ 93.4, 93.4 (C^1, $C^{1'}$), 72.5, 72.2, 71.7, 71.0, 70.9, 70.7, 69.6 (C^2, C^3, C^5, $C^{2'}$, $C^{3'}$, $C^{4'}$, $C^{5'}$), 61.9 (C^4-N_3), 60.6, 60.5 (C^6, $C^{6'}$). *m/z* (ES^+): $[M+Na]^+$ calcd. for $C_{12}H_{21}O_{10}N_3Na^+$, 390.1; found 390.1.

α-D-galactopyranosyl-(1→1)-α-D-glucopyranoside: galactotrehalose

2,3,6,-tri-*O*-benzoyl-α-D-galactopyranosyl-(1→1)-2′,3′,4′,6′,-tetra-*O*-benzoyl-α-D-glucopyranoside (663 mg, 0.619 mmol) was dissolved in 0.2 M methanolic NaOMe (6.6 mL) and the reaction was stirred at room temperature for 14 h. Amberlite® IR120 acidic resin (200 mg) was then added and the mixture stirred at room temperature for 30 mins to neutralise the reaction, filtered and concentrated *in vacuo*. The residue was taken up in water (20 mL), washed with petroleum ether (40-60 °C) and then lyophilised. The crude product was purified by column chromatography (1:4:4 water/EtOAc/isopropanol) to give the product as a white solid (177 mg, 83 %); ^{1}H NMR (400 MHz, D_2O) δ 5.13 (2H, dd, *J* = 6.0, 4.0 Hz, H^1, $H^{1'}$), 3.99 (1H, dd, *J* = 7.0, 5.5 Hz), 3.90 – 3.96 (2H, m), 3.82 (1H, dd, *J* = 10.0, 4.0 Hz), 3.60 – 3.80 (6H, m), 3.57 (1H, dd, *J* = 10.0, 4.0 Hz), 3.36 (1H, t, *J* = 9.5 Hz); ^{13}C NMR (100 MHz, D_2O) δ 93.3, 93.1 (C^1, $C^{1'}$), 72.5, 72.1, 71.3, 71.1, 69.7, 69.3, 68.9, 67.9 (C^2, C^3, C^4, C^5, $C^{2'}$, $C^{3'}$, $C^{4'}$, $C^{5'}$), 61.19, 60.50 (C^6, $C^{6'}$); *m/z* (ES^-): $[M-H]^-$ calcd. for $C_{12}H_{21}O_{11}^-$, 341.3; found 341.2.

Chemoenzymatic synthesis of α-D-mannopyranosyl-(1→1)-α-D-glucopyranoside: mannotrehalose

Trehalose synthase (TreT) enzyme from *Thermoproteus tenax* was overexpressed and purified as described previously (4). The enzymatic reaction contained D-mannose (45 mM), UDP-glucose (30 mM), $MgCl_2$ (40 mM), and TreT (300 µg/mL) in 50 mM HEPES, 300 mM NaCl, pH 8.0 and was incubated at 70 °C for 2 h, with shaking (300 rpm). Reactions were quenched by the addition of an equal volume of cold acetone, cooled

at -20 °C for 1 h and centrifuged (18,000 *g*, 4 °C, 20 min). The supernatant was collected and concentrated *in vacuo*, resuspended in water and purified by size exclusion using Biogel-P2 column (Biorad) using water as an eluent and further purified using HPLC (amino-column: 5 µm, 250 x 4.6 mm (Phenomenex Luna) at 40 °C; mobile phase: 80 % acetonitrile in water, flow rate: 2.5 mL/min; detection: refractive index). The product was obtained as a white solid (6.1 mg, 59.5 %). ^{1}H NMR (400 MHz, D_2O) δ_{ppm} 5.10 (1H, d, *J* = 3.5 Hz, H^1), 5.04 (1H, d, *J* = 1.5 Hz, $H^{1'}$), 3.91 (1H, dd, *J* = 3.5, 2.0 Hz), 3.86 (1H, dd, *J* = 9.5, 3.5 Hz), 3.74 – 3.83 (2H, m), 3.63 – 3.74 (4H, m), 3.52 – 3.63 (3H, m), 3.35 (1H, t, *J* = 9.5 Hz). ^{13}C NMR (100 MHz, D_2O) δ_{ppm} 95.0, 93.5 (C^1, $C^{1'}$), 73.2, 72.6, 72.4, 70.9, 70.2, 70.0, 69.6, 66.7 (C^2, C^3, C^4, C^5, $C^{2'}$, $C^{3'}$, $C^{4'}$, $C^{5'}$), 60.9, 60.5 (C^6H_2OH and $C^{6'}H_2OH$). *m/z* (ES^-): $[M-H]^-$ calcd. for $C_{12}H_{21}O_{11}^-$, 341.3; found 341.1.

Chemoenzymatic synthesis of 3-azido-3-deoxy-α,α′-trehalose

Trehalose synthase (TreT) enzyme from *Thermoproteus tenax* was overexpressed and purified as described previously (4,5). The enzymatic reaction contained 3-azido-3-deoxy-D-glucopyranose (30 mM), UDP-glucose (45 mM), $MgCl_2$ (40 mM), and TreT (300 µg/mL) in 50 mM HEPES, 300 mM NaCl, pH 8.0 and was incubated at 70 °C for 2 h, with shaking (300 rpm) and then cooled by placing on ice. A 10 kDa centrifugal filter unit was prerinsed with 2 mL of deionised water three times by centrifugation at 3200 x g for 20 mins. The cooled enzymatic reaction was then added and centrifuged at 3200 x g for 20 mins, after which the centrifuge filter was washed 3 times with 2 mL deionised water (3200 x g, 20 mins). The filtrates were combined, Bio-Rad Bio-Rex RG 501-X8 resin (1 g) added and the mixture stirred at room temperature for 1 h. The mixture was filtered and the resin washed with 10 mL deionised water. The filtrates were combined and lyophilised to give the product as a white solid (8.7 mg, 53 %). ^{1}H NMR (400 MHz, D_2O) δ 5.12 (1H, d, *J* = 4.0 Hz, H^1), 5.10 (1H, d, *J* = 3.5 Hz, $H^{1'}$), 3.63 – 3.81 (8H, m), 3.55 – 3.61 (2H, m, H^2, $H^{2'}$), 3.42 (1H, t, *J* = 10.0 Hz), 3.36 (1H, t, *J* = 9.5 Hz). ^{13}C NMR (100 MHz, D_2O) δ 93.2, 92.7 (C^1, $C^{1'}$), 72.5, 72.2, 71.0, 69.8, 69.6, 68.5, 65.5 (C^2, C^4, C^5, $C^{2'}$, $C^{3'}$, $C^{4'}$, $C^{5'}$), 62.4 (C^3-N_3), 60.45, 60.2 (C^6, $C^{6'}$). *m/z* (ES^+): $[M+Na]^+$ calcd. for $C_{12}H_{21}O_{10}N_3Na^+$, 390.1; found 390.1.

Chemoenzymatic synthesis of *N*-acetyl-2-amino-2-deoxy- α,α′-trehalose (TreNAc)

Trehalose synthase (TreT) enzyme from *Thermoproteus tenax* was overexpressed and purified as described previously (4). The enzymatic reaction contained D-glucose (45mM), UDP-*N*-acetylglucosamine (50 mM), $MgCl_2$ (40mM), and TreT (350 µg/mL) in 50 mM Tris-HCl, 300 mM NaCl, pH 8.0 and was incubated at 70 °C for 2 h, with shaking (300 rpm) and then cooled by placing on ice. A 10 kDa centrifugal filter unit was prerinsed with 2 mL of deionised water three times by centrifugation at 3200 x g for 20 mins. The cooled enzymatic reaction was added and centrifuged at 3200 x g for 20 mins, after which the centrifuge filter was washed 3 times with 2 mL deionised water (3200 x g, 20 mins). The filtrates were combined and Bio-Rad Bio-Rex RG 501-X8 resin (1 g) was added and the mixture stirred at room temperature for 1 h. The mixture was then filtered and the resin washed with 10 mL deionised water. The filtrates were combined and lyophilised to give the product as a white solid (23 mg, 89 %). ^{1}H NMR (400 MHz, D_2O) δ 5.11 (1H, d, *J* = 3.5 Hz, H^1), 5.08 (1H, d, *J* = 4.0 Hz, $H^{1'}$), 3.88 (1H, dd, *J* = 11.0, 3.5 Hz, H^2), 3.65 – 3.85 (7H, m, H^3, $H^{3'}$, H^5, H^{6a}, H^{6b}, $H^{6a'}$,

$H^{6b'}$) 3.57 (1H, dd, *J* = 9.5, 3.5 Hz, $H^{2'}$), 3.35 – 3.51 (3H, m, H^5, H^4, $H^{4'}$), 1.96 (3H, s, CH_3). ^{13}C NMR (100 MHz, D_2O) δ 174.3 (C=O), 93.4, 91.8 (C^1, $C^{1'}$), 72.7, 72.5, 72.3, 70.8, 70.0, 70.0, 69.3 ($C^{2'}$, C^3, $C^{3'}$, C^4, $C^{4'}$, C^5, $C^{5'}$), 60.5, 60.1 (C^6, $C^{6'}$), 53.5 (C^2), 21.7 (CH_3). *m/z* (ES^+): $[M+Na]^+$ calcd. for $C_{14}H_{25}NO_{11}Na^+$, 406.1; found 406.2.

2-Azido-2-deoxy-α,α′-trehalose

N-Acetyl-2-amino-2-deoxy- α,α′-trehalose (23 mg, 0.060 mmol) was dissolved in hydrazine hydrate solution (50-60%, 10 mL) and stirred under nitrogen at 100 °C for 5 days. The reaction mixture was concentrated *in vacuo* and the residue purified by column chromatography (5:3:2 *n*-butanol/ethanol/water) to give 2-amino-2-deoxy-α,α′-trehalose as a white solid (21 mg). This was dissolved in water (80 µL) then K_2CO_3 (16 mg, 0.115 mmol) and $CuSO_4.5H_2O$ (100 µL of a 6.7 mg/mL solution, 0.0027 mmol) were added. Freshly prepared TfN_3 (approximately 0.27 mmol, in approximately 2 mL DCM) was then added followed by methanol (1 mL) to give homogeneity (6). The reaction mixture was stirred at room temperature for 18 hours, and then concentrated *in vacuo.* The residue was purified by column chromatography ($CHCl_3$/MeOH 1.5:1) to give 2-azido-2-deoxy- α,α′-trehalose as a white solid (17 mg, 77%). ^1H NMR (400 MHz, D_2O) δ 5.23 (1H, d, *J* = 3.5 Hz, H^1), 5.12 (1H, d, *J* = 4.0 Hz, $H^{1'}$), 3.98 (1H, t, *J* = 10.0 Hz, H^3), 3.61 – 3.84 (7H, m, $H^{3'}$, H^5, $H^{5'}$, H^{6ab}, $H^{6'ab}$), 3.56 (1H, dd, *J* = 10.0, 4.0 Hz, $H^{2'}$), 3.45 (1H, t, *J* = 9.5 Hz, H^4), 3.39 (1H, t, *J* = 9.5 Hz, $H^{4'}$), 3.34 (1H, dd, *J* = 10.5, 4.0 Hz, H^2). ^{13}C NMR (100 MHz, D_2O) δ 93.4, 92.6 (C^1, $C^{1'}$), 72.7, 72.7, 72.4, 70.8, 70.5, 69.8, 69.5 (C^2, C^3, $C^{3'}$, C^4, $C^{4'}$, C^6, $C^{6'}$) 62.4 ($C^{2'}$-N_3), 60.5, 60.4 (C^6, $C^{6'}$). *m/z* (ES^+): $[M+Na]^+$ calcd. for $C_{12}H_{21}O_{10}N_3Na^+$, 390.1; found 390.1.

References

1. Sievers, F., Wilm, A., Dineen, D., Gibson, T. J., Karplus, K., Li, W., Lopez, R., McWilliam, H., Remmert, M., Soding, J., Thompson, J. D., and Higgins, D. G. (2011) Fast, scalable generation of high-quality protein multiple sequence alignments using Clustal Omega. *Mol Syst Biol* **7**, 539
2. Robert, X., and Gouet, P. (2014) Deciphering key features in protein structures with the new ENDscript server. *Nucleic Acids Res* **42**, W320-324
3. Monaco, S., Tailford, L. E., Juge, N., and Angulo, J. (2017) Differential Epitope Mapping by STD NMR Spectroscopy To Reveal the Nature of Protein-Ligand Contacts. *Angew Chem Int Ed Engl* **56**, 15289-15293
4. Urbanek, B. L., Wing, D. C., Haislop, K. S., Hamel, C. J., Kalscheuer, R., Woodruff, P. J., and Swarts, B. M. (2014) Chemoenzymatic synthesis of trehalose analogues: rapid access to chemical probes for investigating mycobacteria. *ChemBioChem* **15**, 2066-2070
5. Parker, H. L., Tomas, R. M. F., Furze, C. M., Guy, C. S., and Fullam, E. (2020) Asymmetric trehalose analogues to probe disaccharide processing pathways in mycobacteria. *Org Biomol Chem* **18**, 3607-3612
6. Alper, P. B., Hung, S.-C., and Wong, C.-H. (1996) Metal catalyzed diazo transfer for the synthesis of azides from amines. *Tetrahedron Letters* **37**, 6029—6032
